# Safety and efficacy of human gingiva-derived mesenchymal stem cells in patients with type 1 diabetes: a double-blind, randomized, placebo-controlled, phase 1 trial

**DOI:** 10.1038/s41392-026-03021-8

**Published:** 2026-09-30

**Authors:** Qi Tang, Li Zhou, Liwen Fan, Wei Zhang, Xixiang Tang, Donglan Zeng, Mei Li, Yanhua Zhu, Mengyin Cai, Jia Yuan, Zheng Chen, Song Guo Zheng, Yanming Chen

**Affiliations:** 1 Department of Endocrinology and Metabolism, Guangdong Provincial Key Laboratory of Diabetology & Guangzhou Municipal Key Laboratory of Mechanistic and Translational Obesity Research, the Third Affiliated Hospital of Sun Yat-sen University, Guangzhou, China; 2 Department of Clinical Immunology Center, the Third Affiliated Hospital of Sun Yat-sen University, Guangzhou, China; 3 VIP Medical Service Center, the Third Affiliated Hospital of Sun Yat-sen University, Guangzhou, China; 4 Department of Stomatology, the Third Affiliated Hospital of Sun Yat-sen University, Guangzhou, China; 5 Department of Immunology, School of Cell & Gene Therapy, Songjiang Institute and Songjiang Hospital Affiliated to Shanghai Jiao Tong University School of Medicine, Shanghai, China; 6 Department of Endocrinology and Metabolism, the Eighth Affiliated Hospital of Sun Yat-sen University, Shenzhen, China

**Keywords:** Endocrine system and metabolic diseases, Stem cells

## Abstract

Current therapies for type 1 diabetes mellitus (T1DM) rarely halt progressive β-cell function loss due to autoimmune attack. Human gingiva-derived mesenchymal stem cells (GMSCs) have been shown to have therapeutic effects on a variety of autoimmune diseases through their potent immunomodulatory capacity, but clinical evidence for these effects is lacking in patients with T1DM. Here, we performed a double-blind, randomized, placebo-controlled, phase 1 trial to evaluate the safety and efficacy of GMSC therapy in T1DM patients (ChiCTR1800018339). Patients received 4 intravenous injections of GMSCs (1.0 × 10^6^/kg) or placebo at intervals of 3 weeks between each treatment in combination with routine insulin therapy during the entire trial. All 18 randomized patients completed the trial. At week 12, patients receiving GMSCs displayed improvements in residual β-cell function, as evaluated by C-peptide levels, along with a decrease in the requirement for daily insulin treatment. Remarkably, one patient who received GMSC therapy achieved insulin withdrawal after the first treatment. Moreover, both groups had equal glycemic control, and GMSC therapy was not associated with an increased risk of adverse events, including hypoglycemia. Mechanistically, peripheral exhausted CD8^+^ T-cell proportions were significantly increased, which is consistent with the results of single-cell sequencing and in vivo and in vitro experiments, suggesting that GMSCs potentially induce CD8^+^ T-cell exhaustion to alleviate β-cell autoimmune injury. Overall, this study is the first clinical trial evaluating the safety and therapeutic effect of GMSC therapy in T1DM patients, indicating that GMSCs might serve as a promising novel treatment option for T1DM.

## Introduction

Type 1 diabetes mellitus (T1DM) is an autoimmune disease characterized by the progressive destruction of β-cells in the islets of Langerhans. Islets of Langerhans are an endocrine tissue located within the pancreas and mainly consist of insulin-secreting β-cells, glucagon-secreting α-cells, and a small number of endocrine cells that produce other hormones. Patients with T1DM progressively lose β-cell function, resulting in a lifelong dependence on exogenous insulin. In recent decades, continuous technological advances have greatly optimized diabetes management strategies, including intelligent insulin infusion pumps, real-time continuous glucose monitoring systems, and personalized glycemic intervention regimens. Despite the rapid development of these symptomatic treatment approaches, they rarely help block the ongoing autoimmune injury and prevent the progressive degeneration of islets. From the perspective of clinical practice, the progressive decline of C-peptide is irreversible in the natural history of T1DM.^1^ Multiple innate and adaptive immune cell subsets, including antigen-presenting cells, T cells, B cells, macrophages and innate lymphocytes, collaboratively initiate and amplify intra-islet injury.^2^ Nevertheless, accumulating evidence has firmly established that T-cell-mediated autoimmune activity has been shown to be a key factor in islet immune damage.^3^ Persistent overactivation of self-reactive T cells could induce β-cell apoptosis via releasing perforin and granzyme cytotoxic granules and further trigger cell death through Fas/FasL caspase-dependent signaling pathways.^4^ In addition, sustained inflammatory cytokines derived from hyperactivated T cells could trigger endoplasmic reticulum stress and oxidative damage, thereby forming a self-amplifying autoimmune loop that continuously aggravates islet injury.^5^ Thus, alleviating T-cell immune attack on β-cells is a fundamental way to promote the retention of islet function and improve glycemic control.

T-cell exhaustion is a progressive decline in T-cell function due to continued stimulation of T-cell receptors (TCRs) in the presence of sustained antigen exposure. Exhausted T cells are characterized by high coexpression of inhibitory receptors (such as PD-1, TIGIT, CD244, and LAG3), altered metabolism, and impaired proliferative potential and survival.^6^ Current studies suggest that persistent T-cell activation without exhaustion is a vital cause of T1DM pathogenesis^7^ and that facilitating exhaustion effectively ameliorates T1DM progression.^8–10^ Mesenchymal stem cells (MSCs) are multipotent progenitor cells^11^ that can be isolated and expanded from bone marrow,^12^ the umbilical cord,^13^ adipose tissue,^14^ the gingiva,^15^ and other tissues. Owing to their proven immunomodulatory effects, such as inhibition of effector T-cell activation, induction of regulatory T-cell populations, modulation of antigen-presenting cell function, reduction in β-cell apoptosis, and direct secretion of cytokines or exosomes,^16–20^ MSCs have garnered considerable attention for their potential use in autoimmune diseases, including rheumatoid arthritis,^21^ systemic lupus erythematosus, systemic sclerosis,^22^ and ulcerative colitis,^23^ as well as T1DM.

Although MSCs have impressive therapeutic effects in both preclinical and clinical settings, some potential difficulties still exist and hinder their clinical application. The extraction of MSCs from some sources is invasive and low yielding, and the amplification in vitro is slow. In addition, unremovable allogeneic hematopoietic stem cells mixed with MSCs can induce fatal complications in recipients, such as acute graft-versus-host disease, which increase the cost and risk of therapy. In recent years, gingiva-derived MSCs (GMSCs) have attracted increased attention because of their convenience of isolation, rapid expansion in vitro, low immunogenicity and tumorigenesis, and similar or even superior differentiation and immunomodulation capacity to those of other types of MSCs.^15,24^ In a preclinical study, we performed a systemic evaluation in multiple animal models and validated that GMSC administration displayed favorable biocompatibility with no obvious organ toxicity, adverse systemic reactions, genotoxicity, or tumorigenic risk.^25^ Our previous studies have also demonstrated the therapeutic potential of GMSCs in several immune-related diseases, such as arthritis,^26^ osteoporosis,^27^ lupus nephritis,^28^ atherosclerosis,^29^ asthma,^30^ acute glaucoma,^31^ and aplastic anemia,^32^ and have reported that GMSCs ameliorate STZ-induced T1DM in mice by suppressing IL-17 and IFN-γ production in T effector cells and upregulating regulatory T (Treg) cells.^33^ Nevertheless, convincing evidence from rigorous clinical experiments on the therapeutic effect of GMSCs on T1DM is still lacking, and whether GMSCs affect T-cell exhaustion remains to be further explored.

To evaluate the clinical translational potential of GMSCs in T1DM, we conducted this double-blind, randomized, placebo-controlled, phase 1 clinical trial to assess the safety and efficacy of GMSC therapy in patients with T1DM. All safety-related indicators were closely documented following GMSC administration, and C-peptide levels were monitored to evaluate whether GMSC treatment could preserve β-cell function. Combined with single-cell transcriptomic profiling of peripheral blood mononuclear cells (PBMCs), we preliminarily explored the possible underlying immunomodulatory mechanisms of GMSCs in T1DM. Collectively, this study provided rigorous clinical evidence regarding GMSCs therapy for T1DM, and offered a promising new direction for the development of immune-targeted therapies for T1DM.

## Results

### Description of patients

Among the 23 patients who were screened from March 2022 through May 2023, 18 patients were recruited and randomized to receive GMSCs (*n* = 9) or placebo (*n* = 9). All patients completed the study. An intention-to-treat population was included for further analysis (Supplementary Fig. 1).

Baseline demographic and clinical characteristics were generally balanced between the two groups (Table 1). The baseline HbA1c levels of the placebo and GMSC groups were 7.0% [6.9%, 9.0%] and 7.1% [5.9%, 13.2%], respectively, and the C-peptide area under the concentration‒time curve (C-peptide AUC) was 0.04 [0.04, 0.04] nmol/L*h and 0.04 [0.04, 0.19] nmol/L*h, respectively, and the daily insulin dose was 0.54 [0.52, 0.64] IU/kg/day and 0.57 [0.54, 0.70] IU/kg/day, respectively.

**Table 1:** Demographic and clinical characteristics of T1DM patients at baseline

	All^a^	GMSC^a^	Placebo^a^
Age, year	31.39 ± 9.37	29.11 ± 9.41	33.67 ± 9.29
Sex(M/F), *n*	7/11	4/5	3/6
Weight, kg	59.28 ± 8.17	56.72 ± 5.86	61.83 ± 9.64
BMI, kg/m^2^	21.66 ± 2.92	20.01 ± 2.67	23.30 ± 2.21
Duration, years	4.28 ± 2.92	3.78 ± 2.58	4.77 ± 3.31
FBG, mmol/L	5.66 ± 1.22	5.14 ± 1.24	6.18 ± 0.99
2 h-PBG, mmol/L	12.55 ± 5.63	12.79 ± 6.17	12.31 ± 5.41
AUC of BG, mmol/L*h	20.10 ± 7.12	19.73 ± 7.06	20.46 ± 7.60
Fasting C-peptide, nmol/L	0.02 [0.02, 0.03]	0.02 [0.02, 0.05]	0.02 [0.02, 0.02]
Peak C-peptide, nmol/L	0.02 [0.02, 0.06]	0.02 [0.02, 0.16]	0.02 [0.02, 0.02]
AUC of C-peptide, nmol/L*h	0.04 [0.04, 0.11]	0.04 [0.04, 0.19]	0.04 [0.04, 0.04]
Daily insulin dose, IU/d/kg	0.57 [0.52, 0.67]	0.57 [0.54, 0.70]	0.54 [0.52, 0.64]
HbA1c, %	7.05 [6.40, 9.58]	7.10 [5.90, 13.20]	7.00 [6.90, 9.00]
GA, %	19.98 ± 8.83	21.15 ± 11.37	18.94 ± 6.35
ICA, COI	0.16 [0.08, 0.40]	0.16 [0.08, 0.44]	0.24 [0.08, 0.39]
ICA positive/negative, *n*	3/15	2/7	1/8
IAA, COI	0.50 [0.14, 2.06]	0.28[0.24,4.59]	1.27 [0.14, 1.93]
IAA positive/negative, *n*	7/11	3/6	4/5
GADA, IU/ml	2.76 [1.30, 19.10]	1.81 [1.30, 11.50]	3.09 [1.41, 20.00]
GADA positive/negative, *n*	8/10	4/5	4/5
IA2, IU/ml	0.70 [0.70, 1.21]	0.80 [0.70, 1.86]	0.70 [0.70, 0.93]
IA2 positive/negative, *n*	1/17	1/8	0/9
ALT, U/L	13.0 [10.25, 17.75]	16.00 [11.00, 18.00]	12.00 [10.00, 16.00]
AST, U/L	18.28 ± 5.86	18.00 ± 3.91	18.56 ± 7.58
Cr, umol/L	55.27 ± 9.38	55.99 ± 9.24	54.54 ± 10.02
BUN, mmol/L	5.13 ± 1.20	5.32 ± 0.81	4.94 ± 1.52
TC, mmol/L	4.92 ± 0.91	4.89 ± 0.81	4.95 ± 1.05
TG, mmol/L	0.77 ± 0.28	0.64 ± 0.12	0.89 ± 0.35
LDL-C, mmol/L	2.73 ± 0.73	2.74 ± 0.68	2.72 ± 0.81
HDL-C, mmol/L	1.45 ± 0.31	1.44 ± 0.25	1.46 ± 0.38
24 h uALB, g	13.52 ± 7.08	16.61 ± 8.57	10.04 ± 2.11
ACR, mg/g	7.33 [6.48, 10.10]	9.72 [6.79, 10.74]	6.61 [6.46, 8.51]
UA, umol/L	299.0 [280.8, 416.3]	288.0 [280.0, 399.0]	322.0 [298.0, 422.0]

*BMI* body mass index, *FBG* fasting blood glucose, *2* *h-PBG* two-hour postprandial blood glucose, *HbA1c* glycated hemoglobin, *GA* glycated albumin, *ICA* islet cell antibody, *IAA* insulin autoimmune antibodies, *GADA* glutamic acid decarboxylase antibody, *IA2* insulin-associated-2 antibody, *Cr* creatinine, *BUN* blood urea nitrogen, *TC* total cholesterol, *TG* triglyceride, *LDL-C* low-density lipoprotein cholesterol, *HDL-C* high-density lipoprotein cholesterol, *24* *h uALB* 24-h urine albumin, *ACR* urinary albumin to creatinine ratio, *UA* uric acid

^a^Results are presented as the mean ± SD or median [IQR] according to normal distribution status

### Efficacy end point over the course of the study

**Fig. 1:**
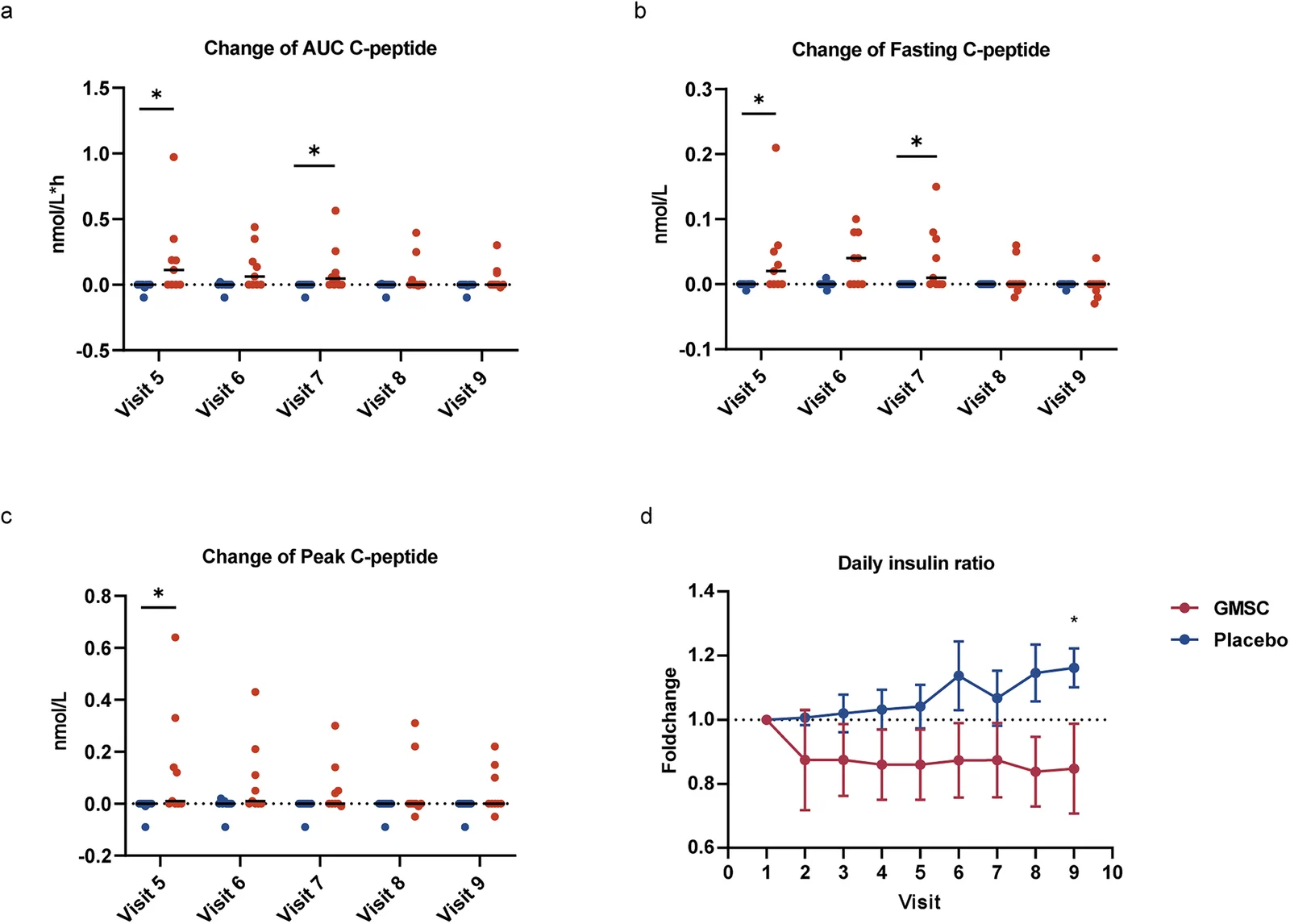
Efficacy end points over the course of the study. Changes in **a** the area under the C-peptide curve, **b** fasting C-peptide level, and **c** peak C-peptide level from baseline in the GMSC group and placebo group during the study. Since the lower limit of the detection range for C-peptide levels at our research center was 0.02 nmol/L, when a patient’s C-peptide level fell below this detection limit, the minimum value of 0.02 nmol/L was assigned. The short lines represent the median values. **d** Fold change in the daily insulin dose compared with baseline in the GMSC group and placebo group during the study. The data are presented as the mean ± SEM. *P < 0.05

To evaluate the β-cell function of the participants, the C-peptide AUC, fasting C-peptide level and peak C-peptide level were strictly measured during a 2-h mixed-meal tolerance test. At week 12, a consistent upward trend in the C-peptide AUC, fasting C-peptide level and peak C-peptide level was observed in the GMSC-treated group compared with those in the placebo control group, with between-group differences reaching statistical significance (*P* < 0.05; Fig. 1a–c; Supplementary Fig. 2a–c). Notably, more than half of the patients (55.6%, 5/9) who received GMSC therapy displayed elevated β-cell function. In contrast, C-peptide levels in the placebo control group remained persistently below the lower limit of detection throughout the study duration.

Moreover, patients in the placebo group progressively required increasing amounts of exogenous insulin to maintain blood glucose stability (Fig. 1d). However, the daily insulin dose of the GMSC group decreased after the first treatment and remained consistently lower than baseline throughout the 48 weeks of the trial (Fig. 1d).

Despite the reduced daily insulin dose in the GMSC group, comparable control of blood glucose was achieved in the GMSC group. There were no differences in the glycemic indices, including fasting glucose levels, postprandial glucose levels, AUCs of glucose levels, and HbA1c levels, between the two groups (Supplementary Fig. 3a–d). The time in range (TIR) index of the GMSC group reached more than 80% (Supplementary Fig. 3e), and the daily glycemic variability index tended to decrease after treatment (Supplementary Fig. 3f–h), suggesting that GMSC therapy could maintain glucose levels within an ideal range without large fluctuations. Notably, one of the patients in the GMSC therapy group achieved insulin withdrawal after the first treatment and was able to maintain a desirable level of glycemic control during the subsequent treatment period (Supplementary Table 1). The fold change in autoantibody levels from baseline was not significantly different between the GMSC and placebo groups (Supplementary Fig. 3i). Only a negative association between the fold change in the islet cell antibody (ICA) level and the change in fasting C-peptide at visit 9 was found in the GMSC group (*Spearman*’s *r* = −0.74; *P* = 0.02). No other association between antibody dynamics and C-peptide preservation was observed, suggesting that the therapeutic effects of GMSCs may depend less on the modulation of humoral autoimmunity.

### Safety assessment

There were no differences in the occurrence of varying degrees of hypoglycemic events between the GMSC and placebo groups over the course of the 48-week trial (Table 2). Some individual patients reported adverse events, including headache, cramps, facial edema, and sleep disorders, but the relationship between these events and GMSC therapy was not statistically significant. These adverse events did not require intervention or medications and regressed well afterward. No serious adverse events were observed, and no adverse events led to study discontinuation. In addition, the clinical laboratory data related to the safety assessment were comparable in both groups (Supplementary Table 2).

**Table 2:** Safety assessment and adverse events

Events	Grade^a^	Person-time	Association with the treatment^b^	Placebo (*n* = 9) *N* (%)	GMSC (*n* = 9) *N* (%)
Hypoglycemia					
<1 event/month	A	/	R	5 (56)	5 (56)
≤1 event/week	B	/	R	2 (22)	3 (33)
≥2 events/week	B	/	R	2 (22)	1 (11)
Headache	A	2	PR	1 (11)	1 (11)
Sore throat	A	1	PIR	1 (11)	/
Limb pain	A	1	PIR	1 (11)	/
Cramps	A	2	PIR	/	1 (11)
Tremor	A	1	PR	1 (11)	/
Sleep disorder	A	1	PIR	/	1 (11)
Tinnitus	A	1	PIR	/	1 (11)
Double vision	A	1	PIR	/	1 (11)
Nocturia	A	1	PIR	1 (11)	/
Facial edema	A	2	PR	1 (11)	/

^a^Grades A to E represent mild, moderate, severe, life-threatening, and death related to adverse events

^b^*R* relevant, *PR* probably relevant, *PIR* probably irrelevant

### GMSCs cause CD8^+^ T-cell exhaustion in patients with type 1 diabetes

**Fig. 2:**
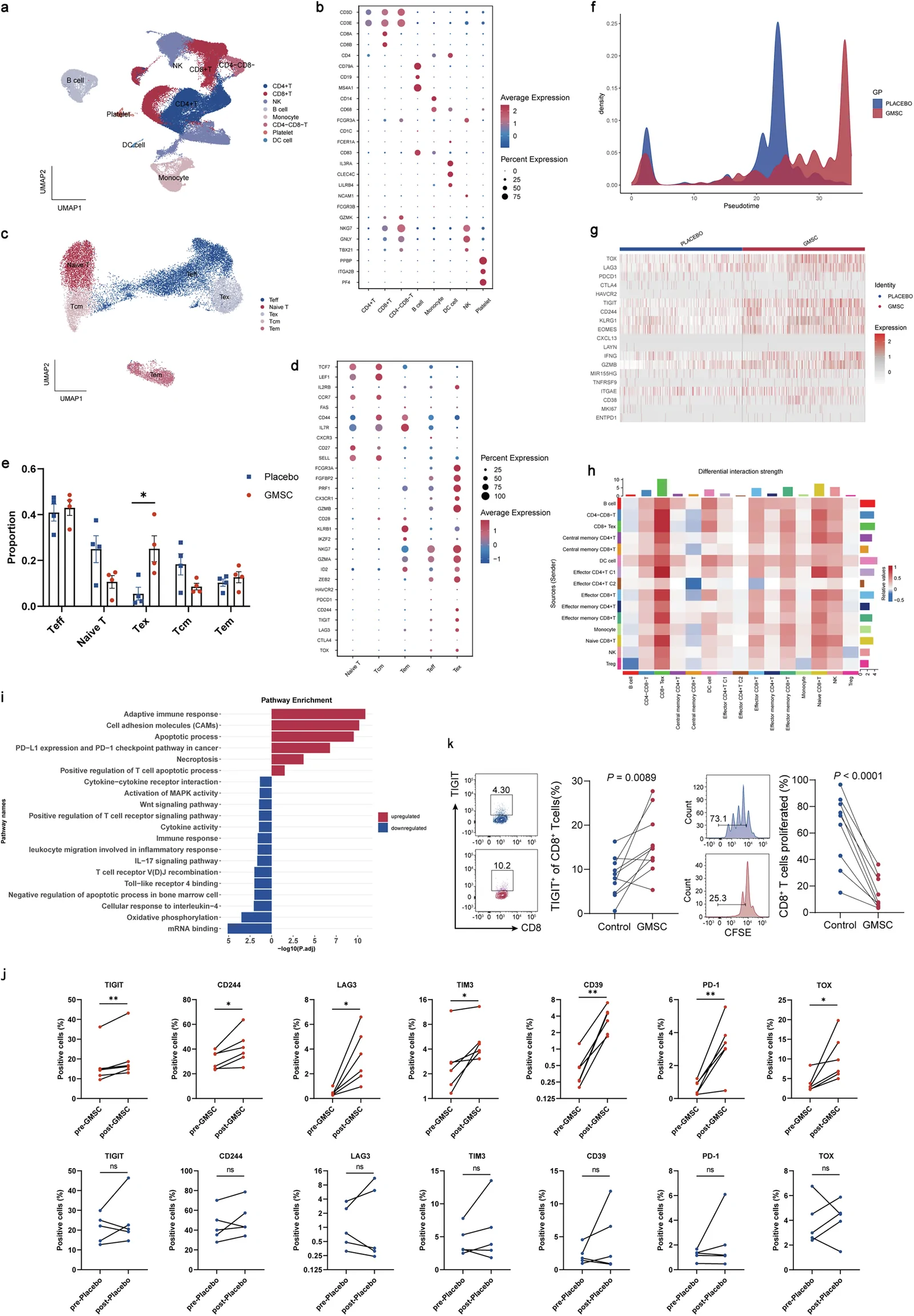
GMSCs cause CD8^+^ T-cell exhaustion in T1DM patients. **a** UMAP visualization of single cells of total PBMCs in gene expression space. Clustering revealed 8 major immune compartments: natural killer (NK), CD8^+^ T, CD4^+^ T, monocyte, B cell, CD8^−^CD4^−^ T, DC, and platelet. **b** Marker genes of the 8 clusters in (**a**). **c** CD8^+^ T cells were clustered individually, and 5 subpopulations were identified: Teff, effective CD8^+^ T cells; Tcm, central memory CD8^+^ T cells; Naive T cells; Tex, exhausted CD8^+^ T cells; and Tem, effective memory CD8^+^ T cells. **d** Marker genes of the 5 clusters of (**c**). **e** The proportions of the subpopulations of CD8^+^ T cells. **f** Pseudotime analysis of CD8^+^ T cells. **g** Heatmap showing the expression of genes related to exhaustion. **h** Heatmap showing the comparison of intercellular communication strength between the GMSC group and the placebo group as assessed by CellChat (ver 2.1.2). The sender cells of the signaling pathway are displayed in rows, and the signal-receiving cells are displayed in columns. Red represents the GMSC group > the placebo group, while blue represents the GMSC group < the placebo group. **i** Pathway enrichment analysis of the differentially expressed genes based on the KEGG and GO databases. **j** Flow cytometry of PBMCs before and after GMSC or placebo injection. **k** PBMCs freshly isolated from patients with T1DM were cocultured with GMSCs, and CD8^+^ T-cell exhaustion and proliferation capacity were assessed. *P < 0.05. **P < 0.01. ns not significant

We randomly selected 8 patients (4 with GMSCs and 4 with placebo) for single-cell RNA transcriptional sequencing (scRNA-seq) of PBMCs at visit 5. We projected approximately 81464 high-quality cells into uniform manifold approximation and projection (UMAP) space, which revealed eight distinct clusters based on specific markers (Fig. 2a, b). GMSC treatment did not affect the global distribution of PBMCs (Supplementary Fig. 4a, b). We further subclustered CD8⁺ T cells, CD4⁺ T cells, NK cells, and monocyte subsets. Although CD4^+^ Treg cells were previously reported to be associated with GMSC therapy, the proportion of Treg cells did not significantly shift after GMSC infusion (Supplementary Fig. 4c–e). The expression levels of canonical Treg phenotypic markers and key genes mediating regulatory capacity were largely equivalent between the two groups (Supplementary Fig. 4f). The strength of ligand‒receptor crosstalk between Tregs and other immune cell populations remained comparable across the two groups (Supplementary Fig. 4g). Similarly, we observed no marked differences in the proportions of proinflammatory and anti-inflammatory monocyte subsets (Supplementary Fig. 5a–c) or CD56^dim^ and CD56^bright^ NK cell subsets (Supplementary Fig. 5e–g). The expression profiles of relevant inflammatory cytokines and functionally critical genes in monocyte subsets (Supplementary Fig. 5d) and NK cell subsets (Supplementary Fig. 5h) were largely comparable between the GMSC and placebo groups.

We then further analyzed CD8^+^ T-cell subsets, and five clusters were identified: effective CD8^+^ T cells (Teffs), naive CD8^+^ T cells (naive T cells), central memory CD8^+^ T cells (Tcms), effective memory CD8^+^ T cells (Tems), and exhausted CD8^+^ T cells (Tex) (Fig. 2c, d, Supplementary Fig. 6a, b). The GMSC recipients had significantly increased Tex proportions (Fig. 2e). Pseudotime analysis confirmed the differentiation trajectory of CD8^+^ T cells (Supplementary Fig. 6c), and GMSC therapy promoted CD8^+^ T-cell progression toward the terminal end of differentiation (Fig. 2f), with a significantly elevated exhaustion score (Supplementary Fig. 6d). Consistently, the expression of exhaustion-related genes, including *TIGIT*, *CD244*, *EOMES*, *LAG3*, and *TOX*, was also upregulated in the GMSC group (Fig. 2g), resulting in immunosuppressive signaling between other immune cells and CD8^+^ T cells, such as the CD160/BTLA-TNFRSF14 signal, in the GMSC group (Supplementary Fig. 6e). Compared with those in the placebo group, Tex cells in the GMSC group received stronger intercellular communication signals (Fig. 2h). Pathway enrichment analysis suggested that multiple proinflammatory pathways and T-cell receptor signaling pathways were suppressed in the GMSC group (Fig. 2i). On the other hand, pathways related to the adaptive immune response, T-cell apoptotic process and immunosuppressive pathway were enhanced in the GMSC group (Fig. 2i). Flow cytometry of PBMCs sorted before and after GMSC treatment also revealed increases in exhausted CD8^+^ T cells marked by positive expression of TIGIT, CD244, LAG3, TIM3, CD39, PD-1, and TOX (Fig. 2j). Coculture of PBMCs with GMSCs in vitro additionally validated the GMSC-mediated induction of T-cell exhaustion and inhibition of T-cell proliferation (Fig. 2k).

### GMSCs promote the conservation of β-cells in a NOD mouse model

**Fig. 3:**
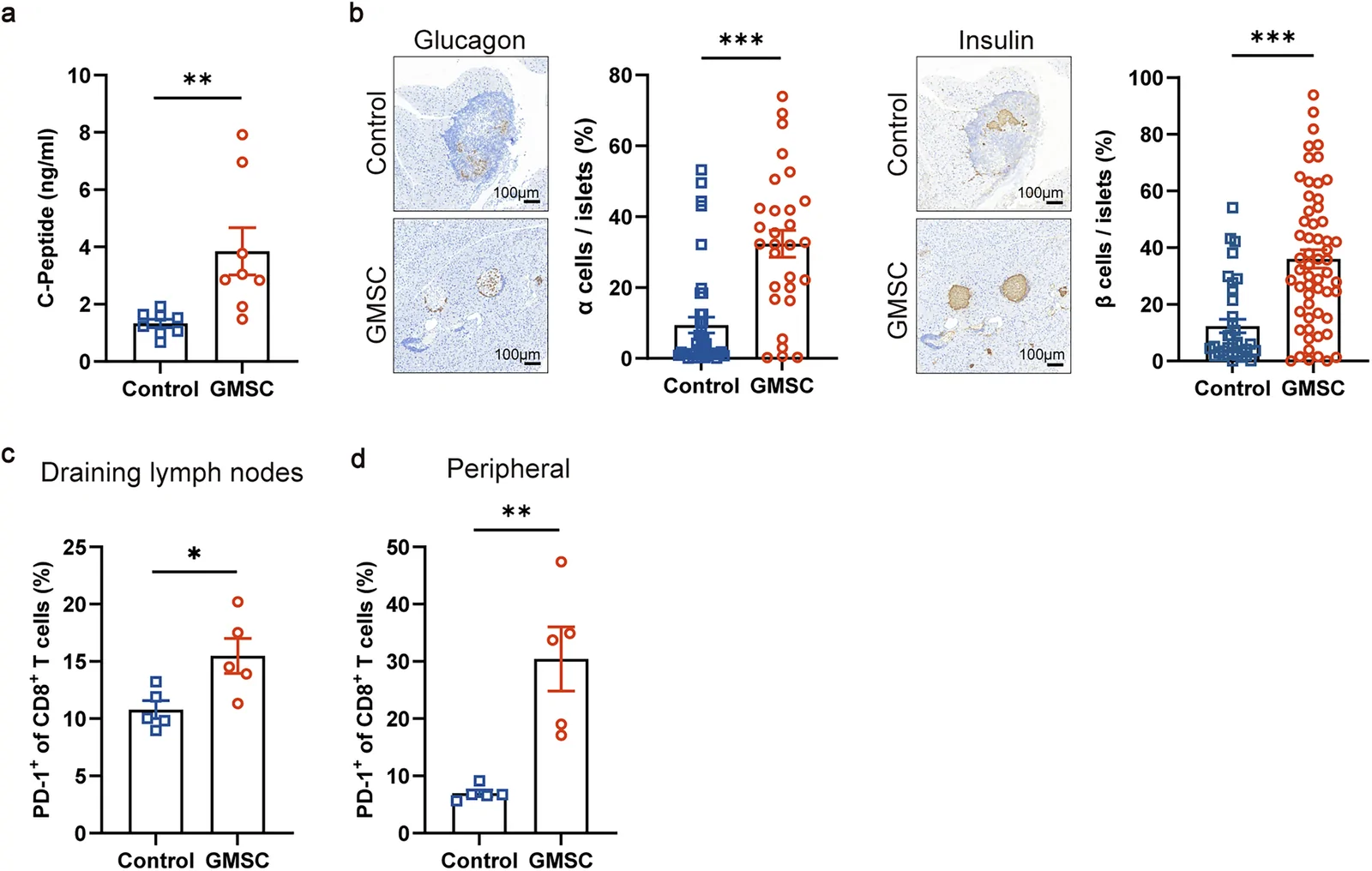
GMSCs promote β-cell conservation in a NOD mouse model. **a** Plasma C-peptide levels in GMSC-treated mice and control mice. **b** Histochemical analyses of mouse pancreatic tissue sections. The α-cell area (positive glucagon staining) and β-cell area (positive insulin staining) were calculated in relation to the islet area. Proportion of exhausted CD8^+^ T cells in **c** draining lymph nodes and **d** peripheral blood of mice. *P < 0.05. **P < 0.01. ***P < 0.001

To directly observe the protective effects of GMSC treatment on pancreatic β-cells, we utilized a NOD mouse model. Following tail vein injection of GMSCs in NOD mice with established T1DM, a significant increase in plasma C-peptide levels was observed compared with those in the control group (Fig. 3a). To clarify the protective effects of GMSCs on β-cells, histochemical analyses were performed. When all the islets were taken as individual samples, compared with control mice, GMSC-treated mice exhibited significantly greater conservation of insulin-producing β-cells per islet (Fig. 3b). Consistent with the observations in patients, there was a significant increase in the proportion of exhausted CD8^+^ T cells in both the draining lymph nodes (Fig. 3c) and the peripheral blood (Fig. 3d) of the GMSC-treated mice.

## Discussion

This randomized, placebo-controlled phase I trial provides preliminary evidence that four intravenous administrations of GMSCs might improve β-cell function in patients with T1DM, as we observed an increasing trend in C-peptide AUC, peak C-peptide, and fasting C-peptide levels in the GMSC group relative to those in the control group, which suggests at least a potential partial recovery of endogenous insulin secretory capacity.

In the placebo group, the daily insulin requirements increased because of the progressive loss of endogenous insulin production, which is consistent with the findings of many previous studies.^34^ However, participants receiving GMSC treatment tended toward attenuated insulin dose escalation and sustained a lower dose of daily insulin for up to one year after therapy alongside stable glycemic control. Notably, one patient discontinued exogenous insulin early in the trial and maintained normoglycemia throughout the study period. However, this individual clinical response remains anecdotal, and we will continue long-term follow-up of this patient to further explore the commonalities and unique individual characteristics distinguishing this case from other enrolled patients in the future. In line with the findings of our study, MSCs from different origins were reported to reduce insulin requirements by approximately 30–50% in patients with either type 1 diabetes or type 2 diabetes over a longer period of intervention.^35,36^ The safety assessment did not identify any safety concerns attributable to GMSC therapy during the 48-week trial period. However, owing to the time constraints of our study, the long-term safety profile of GMSCs warrants further investigation in larger, extended-duration studies.

To elucidate the potential mechanism underlying the therapeutic effect of GMSCs, we performed scRNA-seq on patient PBMCs. During the immune response, a fraction of effector T cells gradually differentiate into exhausted T cells upon persistent antigen stimulation, which serves to prevent excessive immune activation. Pseudotime analysis confirmed that GMSC treatment accelerated the progression from effector CD8^+^ T cells to exhausted CD8^+^ T cells, which was consistent with the increased proportion of exhausted T cells observed in the subgroup analysis. Further analysis revealed that GMSC treatment significantly upregulated the expression of canonical exhaustion-related genes, including *TIGIT* and *CD244*, in CD8^+^ T cells. TIGIT competitively inhibits the binding of the costimulatory receptor CD226 on T cells to CD155 expressed on other immune cells^37,38^ and activates ITIM-dependent signaling, thereby suppressing PI3K/AKT/mTOR-mediated cytokine production^39^ and upregulating the exhaustion-associated key transcription factor TOX to drive CD8^+^ T-cell exhaustion.^40^ High CD244 expression on CD8^+^ T cells results in the recruitment of SHP-1/SHP-2 phosphatases because of decreased SAP adapter levels, thereby suppressing T-cell receptor signaling and leading to impaired proliferation, degranulation, and the production of cytotoxic molecules.^41^ Subsequent analyses revealed downregulated inflammatory cytokine pathways and upregulated apoptotic pathways after GMSC therapy, which further supported the above findings. Specifically, the secretion of the proinflammatory cytokine IL-17, which is associated with the development of T1DM,^42,43^ has been demonstrated to be inhibited by TIGIT.^44^ In vitro cellular coculture experiments and an in vivo NOD mouse model also verified the ability of GMSCs to induce CD8^+^ T-cell exhaustion. Considering that previous evidence suggests a potential relationship between impaired T-cell exhaustion and the pathogenesis of T1DM,^7^ we hypothesize that GMSC therapy somehow promotes T-cell exhaustion and weakens autoimmune T-cell attack on pancreatic islets, thus allowing the restoration of β-cell endocrine function. The preservation of insulin-positive β-cell mass within the pancreatic islets of GMSC-treated NOD mice corroborated our hypothesis. In other clinical studies of T1DM, the therapeutic effect was also reported to correlate with increased CD8^+^ T-cell exhaustion.^9,45^ Numerous studies have focused on targeting T-cell exhaustion to explore novel therapeutic strategies for autoimmune diseases.^46^ The immune therapies researched in T1DM thus far can be largely classified as immune cell targeted, cytokine targeted, and immunotolerant.^47^ For example, the anti-CD3 monoclonal antibody teplizumab exhausts autoreactive T cells.^48^ The soluble CTLA-4 analog abatacept blocks the costimulatory signals required for T-cell activation and proliferation.^49^ JAK1/2 inhibitors inhibit CD8 T-cell activation;^50^ cytokine inhibition drugs targeting IL-1,^51^ IL-6,^52^ IL-12^53^ and TNFα^54^ block innate immunity or direct damage to β-cells. Although some of these novel drugs cannot preserve residual β-cell function and face issues such as transient efficacy, associated infection risks, and the induction of cytokine release syndrome or other autoimmune diseases, exploring T1DM treatment from an immunological perspective is an area of intense interest, and strict clinical trials are needed to further confirm the safety and efficacy of immunotherapies for T1DM.

Our study presents several notable strengths. Through a rigorous RCT, we provided preliminary evidence of potential metabolic benefits conferred by GMSC intervention in patients with T1DM. Given its unique advantages, such as easy preparation and low immunogenicity compared with those of other MSCs, our results also provide a new exploratory direction for the translational application of stem cell-based therapeutics for T1DM. T-cell exhaustion may be involved in the pathogenesis of other autoimmune diseases as well as T1DM. Our elucidation of the relationship between GMSCs and T-cell exhaustion suggests a possible novel approach for managing such diseases.

Our trial had certain limitations. Owing to COVID-19 pandemic restrictions, we recruited only 18 patients in our trial, which was probably underpowered to address some end points and weakens the generalizability of the findings to broader T1DM populations. A substantial proportion of baseline C-peptide measurements were at or near the lower detection threshold, which might have impaired the statistical power of our efficacy evaluation. Larger-scale, longer-term cohort studies recruiting participants with diverse residual β-cell functions are needed in the future to validate our conclusions and improve the generalizability of the results. Second, although prior published evidence indicated that the induction of CD8⁺ T-cell exhaustion could mitigate disease progression in patients with T1DM, our human trial revealed only a concurrent increase in CD8⁺ T-cell exhaustion and improved β-cell function, which could not serve as definitive causal evidence. We plan to conduct more experiments to formally verify the causal relationship between CD8⁺ T-cell exhaustion and β-cell protection. Third, T-cell exhaustion might limit the anti-tumor therapeutic effect of immune checkpoint inhibitors; thus, the use of GMSCs in T1DM patients with comorbid tumors awaits further study. In addition, the specific mechanism by which GMSCs induce CD8^+^ T-cell exhaustion remains unclear. Researchers have previously reported roles for both cell‒cell contact and the release of soluble factors, such as prostaglandin E2, indoleamine-2,3-dioxygenase, nitric oxide, interleukin-27, transforming growth factor-beta, monocyte chemotactic protein 1, and human leukocyte antigen G, in MSC-mediated T-cell suppression.^55–57^ Our further study is in progress to determine the potential candidate mediators involved in GMSC-T-cell exhaustion interactions. Some studies have also reported that GMSCs can exert other immune regulatory effects across adaptive and innate immune compartments, including the suppression of proinflammatory monocytes, NK cells, and pathogenic Th1/Th17 cells and the induction of protective Tregs and anti-inflammatory M2-like macrophages.^58–60^ Whether this crosstalk plays a role in T1DM remains to be further explored in follow-up investigations.

## Materials and methods

### Trial design and oversight

We conducted this double-blind, randomized, placebo-controlled trial at the Third Affiliated Hospital of Sun Yat-Sen University and screened participants from 2022–03 through 2023–05. Eligible patients were randomly assigned, at a 1:1 ratio, to receive GMSCs or a matching placebo administered intravenously. Independent data monitoring committees reviewed clinical and safety data and provided trial oversight. The protocol and Institutional Review Board (IRB, #2018-10) were used in accordance with the ethical principles of the Declaration of Helsinki and were reviewed by the Ethics Committees of the Third Affiliated Hospital of Sun Yat-Sen University, Guangzhou, Guangdong, China. Informed consent was obtained from all participants. The protocol is available in the Supplementary Appendix.

### Patients

We enrolled male and female patients 18–60 years of age with T1DM who had been diagnosed within ten years before randomization according to the criteria of the 1999 WHO. Eligible patients should have a fasting C-peptide concentration of no more than 0.3 ng/ml. The key exclusion criteria were a body mass index greater than 30 kg/m^2^, insulin requirements greater than 100 U per day, severe hepatic or renal insufficiency with proliferative diabetic retinopathy or macular edema, diseases requiring steroid hormone therapy, major systemic illnesses, and infection. The full inclusion and exclusion criteria can be found in the Supplementary Appendix. A block randomization method (block size = 4) was employed. Using SPSS 24.0 statistical software, random number sequences were generated, and the subjects were assigned to the GMSC group or placebo group at a 1:1 ratio. Allocation concealment was implemented *via* sequentially numbered, opaque, sealed envelopes. The group allocation results were known only to researchers at the Zhongshan Ophthalmic Center of Sun Yat-sen University who were solely responsible for preparing the stem cell and placebo infusions and were not involved in patient recruitment, intervention, follow-up, or data analysis. Both patients and clinical investigators at the Third Affiliated Hospital of Sun Yat-Sen University remained blinded to the group assignments until the end of the trial. Details on randomization and blinding can be found in the Supplementary Appendix.

### Trial procedures

Patients enrolled in our trial were hospitalized at weeks 0, 3, 6, and 9 for receiving a treatment of GMSCs or a matching placebo. The GMSC mixture contained 1.0 × 10^6^ GMSCs per body weight (kg) suspended in 200 ml of saline, and the same volume of pure saline served as a matching placebo. During each treatment, both groups received intravenous treatment following the same protocol, and the solution was packaged in a brown opaque bag to prevent differentiation by appearance. All bags were immediately retrieved and returned to the Zhongshan Ophthalmic Center after each infusion. Visits 5–8 were performed at weeks 12, 18, 24, and 36 by outpatient services during the follow-up phase, and patients were hospitalized again at week 48 for overall assessment. Efficacy and safety assessments were made at every visit according to the prespecified schedule (Supplementary Fig. 1). Trial investigators were instructed to maintain patients’ intensive glycemic control by administering regular subcutaneous insulin injections or the use of an insulin pump to reach the recommended target glucose levels (defined as a fasting blood glucose concentration ranging from 4.4 to 5.6 mmol/L and a postprandial glucose concentration <7.8 mmol/L). Investigators recorded patients’ daily insulin doses and adverse events during each visit on an electronic case report form.

### End points and assessments

The primary end point was the change in patients’ β-cell function from baseline at week 12 (visit 5), which was assessed by the C-peptide AUC, fasting C-peptide levels and peak C-peptide levels during a two-hour mixed-meal tolerance test. Blood samples were collected at 0, 30, 60, and 120 min during the test. The C-peptide AUC was calculated using the trapezoidal rule. The peak C-peptide concentration was defined as the highest C-peptide concentration detected among all the measured time points. The secondary end points were the change in patients’ β-cell function during other follow-up visits (visits 6–9), changes in patients’ daily insulin doses (units per kilogram per day) over 48 weeks, and changes in the glycometabolism index at scheduled visits throughout the trial. An exploratory analysis explored the potential immunological mechanism underlying the therapeutic effect of GMSCs. Safety was assessed through the collection of reported adverse events (AEs), including clinically hypoglycemic events (defined as a blood glucose concentration <3.9 mmol/L), and clinical laboratory data, such as hepatic and renal function and blood lipid levels.

### GMSC isolation, culture and injection preparations

Healthy human gingival tissues (from healthy Asian males or females aged between 20 and 30 years and without any autoimmune or inflammatory diseases) were obtained as remnant or discarded tissues at the Third Affiliated Hospital of Sun Yat-sen University after IRB approval and informed consent were obtained. After strict disinfection of the surgical area, a small piece of gingival tissue attached to the wisdom tooth was immediately placed into a 15-ml centrifuge tube containing PBS supplemented with 5% antibiotics (500 U/ml penicillin and 500 U/ml streptomycin) and then sent to Zhongshan Ophthalmic Center of Sun Yat-sen University for GMSC isolation and expansion under GMP conditions. Gingival tissues were repeatedly rinsed with PBS containing 5% antibiotics in a biological safety cabinet. The tissues were minced into small pieces using sharp surgical scissors and resuspended in 1 ml of serum-free medium (TAKARA, #Y50200) in a 1.5 ml Eppendorf tube. The suspension was transferred to a 25 cm^2^ cell culture flask, and the tissue fragments were evenly distributed across the entire flask surface. Subsequently, 1 ml of serum-free medium was gently added to the flask. The flask was then placed in a CO_2_ incubator for culture at 37 °C with 5% CO_2_, and the cells were designated passage 0 (P0). After 48 h, the culture supernatants were collected and submitted to the Department of Laboratory Medicine, The Third Affiliated Hospital of Sun Yat-sen University, for comprehensive detection of bacteria, fungi, viruses, and other pathogens. Upon confirmation of negative microbiological contamination, the cells were transferred to the cell preparation room for further culture. The cells were seeded at a density of 6 × 10^5^ cells/ml into 15 cm diameter cell culture dishes and cultured under routine conditions in a CO_2_ incubator (37 °C, 5% CO_2_). Upon reaching 85%–90% confluence in the dishes, the cells were digested with TrypLE™ Select at 37 °C and passaged at a ratio of 1:4 into new 15 cm cell culture dishes. The supernatants from passages 1, 2 and 3 (P1, P2, P3) also underwent multiple tests, including the detection of mycoplasma, bacterial, fungal and viral contamination. When P3 cells reached 85%–90% confluence, the cells were cryopreserved in serum-free freezing medium (Yocon, #NC1001) in cryovials stored at −80 °C. For long-term preservation, the cells were transferred to liquid nitrogen.

For injection preparations, cryopreserved GMSCs were rapidly thawed in a metal bath at 37 °C and immediately added to 10 ml of TAKARA serum-free medium. After centrifugation, the cells were resuspended in TAKARA serum-free medium for subsequent cultivation (passage 4). When the cells reached 85%–90% confluence after one passage (passage 5), they were collected, resuspended in 5 ml of normal saline, and centrifuged at 500 × *g* for 5 min for washing. After three washes, the cell concentration and viability were measured, followed by comprehensive quality inspections, including microbiological contamination detection and characterization identification (detailed in the Supplementary Appendix). For qualified cells, the cell viability detected by trypan blue staining should be ≥90%. The proportions of CD90^+^, CD105^+^, and CD73^+^ cells analyzed by flow cytometry should be ≥95%, and the proportions of CD45^+^, CD34^+^, CD11b^+^, CD19^+^, and HLA-DR^+^ cells should not exceed 2%.^11,61^ Qualified cells were finally resuspended in 200 ml of saline and placed in a light-proof brown opaque infusion bag for subsequent intravenous infusion.

### Isolation of peripheral blood mononuclear cells (PBMCs)

During visits 1–4, blood samples were collected twice on the day before and after each injection. During visits 5–9, blood samples were collected only once. PBMCs were isolated from whole blood samples using a density gradient centrifugation method. Blood samples collected in ethylenediaminetetraacetic acid tubes were diluted with an equal volume of phosphate-buffered saline (PBS) without calcium or magnesium. Ficoll density gradient medium (1.077 g/ml) was then added to the diluted blood, and the mixture was carefully layered to ensure that the gradient was not disturbed. The tubes were centrifuged at 800 × *g* for 30 min at 4 °C without the use of a brake to prevent disruption of the density gradient during deceleration. Following centrifugation, the PBMC layer was carefully aspirated from the interphase, taking care to avoid contamination with the underlying erythrocyte and granulocyte layers. The collected PBMCs were then washed twice by centrifugation at 700 × *g* for 6 min at 4 °C and resuspended in PBS. An erythrocyte lysis step was performed to remove residual red blood cells for 5 min. PBMCs were subsequently washed twice, resuspended in cell freezing medium, and preserved at −80 °C until use.

### Single-cell RNA-seq and cell annotation

Single-cell suspensions were prepared from PBMCs. For library generation, a scRNA-seq reagent kit (10x Genomics) was used. CellRanger (ver 5.0.0) was used to process the sequencing data. Data integration and clustering were performed with the R package ‘Seurat’ (ver 4.3.0.1).^62^ Cells with <500 genes, >6000 genes, or a mitochondrial gene ratio >20% were filtered out. The Seurat ‘FindAllMarkers()’ function was utilized to identify genes with higher expression in each cell subpopulation with default parameters. Cluster annotation was based on the expression of marker genes. The R package ‘monocle’ (ver 2.28.0) was applied for cell trajectory analysis and pseudotime assessment. The R package ‘CellChat’ (ver 2.1.2) was used for cellular communication analysis. Addmodule score analysis was performed with the R package ‘Seurat’ (ver 4.3.0.1).

### Mice

Five-week-old NOD/ShiLtJGpt mice were purchased from GemPharmatech Co., Ltd. All the animals were housed in an SPF animal facility in a temperature (72 ± 3 °F)- and air (50 ± 20% relative humidity)-controlled room with a 12 h light/dark cycle and were given a standard diet and tap water. All experiments using mice were performed in accordance with protocols approved by the Institutional Animal Care and Use Committee of Sun Yat-sen University. For the GMSC-treated group, a single dose of 1.0 × 10^6^ GMSCs resuspended in 200 µL of PBS was injected into NOD/ShiLtJGpt mice once per week for 2 weeks. The same volume of PBS served as a matching control.

### Flow cytometry

Flow cytometry analysis was performed using a panel of antibodies (BioLegend) targeting CD45 (#368516), CD3 (#317325), CD8a (#300919), TIGIT (#372704), CD244 (#329516), CD39 (#328206), TOX (#377123), TIM3 (#364805), LAG3 (#369309), and PD-1 (#329908). CD45 was used as the primary gating marker to isolate total leukocytes from PBMCs. CD3, a definitive surface marker for total T lymphocytes, was used to gate CD3^+^ T cells from CD45^+^ leukocytes. CD8a is a canonical surface marker for CD8^+^ T cells and was used to further isolate a subset of CD3^+^CD8a^+^ T cells from total T lymphocytes. TIGIT, CD244, CD39, TOX, TIM3, LAG3 and PD-1 are canonical exhaustion-associated markers that are highly expressed in exhausted T cells.^6^ In this study, we identified exhausted CD8^+^ T cells on the basis of the positive expression of these markers. The results were obtained on a BD FACS Calibur flow cytometer and analyzed using FlowJo.

### Histology and immunohistochemistry

Pancreatic samples were fixed overnight in 10% formalin and embedded in paraffin. All embedding, sectioning, and staining procedures were performed by Servicebio Company (Wuhan, China). To assess insulin and glucagon production in the pancreas, two consecutive sections were stained with antiinsulin (1:1600; Abcam 63820) or anti-glucagon (1:8000; Abcam ab92517) antibodies, and the staining was visualized with antirabbit DAB-HRP (OMap) secondary antibodies; nuclei were counterstained with hematoxylin and a bluing reagent. Area measurement was performed with ImageJ software. For morphometric analysis of the α-cell area (positive glucagon staining) and β-cell area (positive insulin staining), each islet was traced and analyzed manually with ImageJ software. All islets seen on the sections were measured using the “Freehand selections” tool to draw the outline of islets in images with scale bars, and then the images were converted to RGB stacks, followed by automatic thresholding. The area of α-cells or β-cells in relation to the islet area could be read after the “measure” tool was used. Sections were evaluated by at least two different people to avoid subject bias.

### Statistical analysis

Efficacy and safety analyses were conducted in the intention-to-treat population. For missing data, last observation carried forward (LOCF) was used for imputation under the assumption that the data were missing at random (MAR). This approach was justified given the following: (a) a chronic stable disease state for type 1 diabetes and (b) a short study visit interval. No missing data were observed for the primary end point analysis. All the statistical analyses were performed using the R package ‘stats’ in R (ver 4.3.1). The *P*-value was two-sided for all end points, and a *P*-value < 0.05 was considered to indicate statistical significance. If the data within the GMSC group and placebo group under comparison satisfied the assumptions of normality and homogeneity of variance, the independent samples Student’s *t*-test was employed to evaluate the statistical significance of between-group differences in means. If the data within any group under comparison violated the assumption of normality or homogeneity of variance, the nonparametric Wilcoxon rank-sum test was used to evaluate the statistical significance of between-group differences, and median differences were calculated by the Hodges–Lehmann test. All the statistical analyses were unadjusted. For adverse events, Fisher’s exact test was used to evaluate the occurrence. The statistical analysis plan is available in the Supplementary Appendix.

## Supplementary information

Supplementary Materials

Statistical Analysis Plan

Study Protocol

## Data Availability

The datasets generated during and analyzed in the current study are available from the corresponding author upon reasonable request. scRNA-seq data were deposited in the China National Center for Bioinformation under accession number PRJCA068965 (https://www.cncb.ac.cn/).
