# Supplementary material for: Safety and efficacy of human gingiva-derived mesenchymal stem cells in patients with type 1 diabetes: a double-blind, randomized, placebo-controlled, phase 1 trial
Source: Signal Transduct Target Ther. 2026 Sep 30;11:434. doi: 10.1038/s41392-026-03021-8 (PMC13627707; doi:10.1038/s41392-026-03021-8)
Supplement: Supplementary file 1 [file 41392_2026_3021_MOESM2_ESM.pdf]

Version number: 20220123
Version date: January 23,2022

Statistical Analysis Plan

Clinical trial name: Human gingival mesenchymal stem cell therapy for type 1 diabetes mellitus
Clinical trial participant unit: The Third Affiliated Hospital of Sun Yat-sen University
Principal investigator: Professor Yanming Chen / Song Guo Zheng
Data management and statistical analysis: School of Public Health, Sun Yat-sen University
Trial registration number： chictr.org.cn numb, 1800018339
Statistical director: Professor Ling Li
SAP authors： Professor Ling Li, Dr. Feng Chen, Dr. Qi Tang, Dr. Li Zhou, Dr. Liwen Fan

Sponsor: The Third Affiliated Hospital of Sun Yat-sen University
Person in charge: Professor Yanming Chen
Contact number: 18922102818
Address of the Sponsor: NO.600 Tianhe Road, Tianhe District, Guangzhou City
Zip Code:510630

Content

1. Introduction 3
2. Study objectives 4
3. Trial Design 4
    3.1. Inclusion criteria: 4
    3.2. Exclusion criteria: 5
    3.3. Randomization and blinding 5
    3.4. Sample size 5
4. Variables 5
    4.1. Efficacy variables 6
        4.1.1. Primary endpoint 6
        4.1.2. Secondary endpoints 6
        4.1.3. Exploratory endpoints 6
    4.2. Safety variables 7
        4.2.1. Adverse events (AEs) 7
        4.2.2. Laboratory data related to safety evaluation 7
5. Statistical Consideration 8
    5.1. Timing of analyses 8
    5.2. Study population 8
        5.2.1. Efficacy population 8
        5.2.2. Safety population 8
    5.3. Missing data 8
        5.3.1. Missing data for primary endpoints 8
        5.3.2. Missing data for secondary endpoints 8
        5.3.3. Missing data for date and time of treatment and visiting 8
    5.4. The general statistical principles 9
    5.5. Demographics and baseline characteristics 9
    5.6. Analysis of primary endpoints 10
    5.7. Analysis of secondary endpoints 10
    5.8. Analysis of exploratory endpoints: 10
    5.9. Analysis of safety data 10
        5.9.1. Analysis of adverse events 10
        5.9.2. Analysis of laboratory data 12

1. Introduction

The cost of diabetes treatment is staggering, accounting for 12% of total global health spending. The latest data shows that the prevalence of diabetes in Chinese adults is 11.0%, and the total number of patients is 113.9 million. Among them, 7%-12% are type 1 diabetes, and the prevalence is still growing at a rate of 3% per year. Type 1 diabetes (T1DM) is an autoimmune disease that occurs in genetically susceptible individuals, which is mediated by T cells. The islet β-cell function is deficient and/or reduced in capacity, resulting in insulin secretion not meeting the body's needs. Currently, it relies mainly on insulin replacement therapy. This treatment cannot accurately simulate the regulation of glucose homeostasis by islet β cells, and as the disease progresses, various complications will gradually appear, which has a high lethal morbidity rate. Actively exploring ways to improve or repair islet β-cell damage is of great significance for opening up new areas of diabetes treatment. Maintaining or rebuilding islet function by various means has always been a research hotspot in the field of diabetes treatment. Although pancreatic or islet transplantation has made great progress in establishing endogenous insulin secretion system, the donor organ is seriously deficient, and islet cells are low *in vitro* survival rates and post-transplant immune rejection severely limit their clinical application.

In recent years, the rapid development of stem cell theory and technology has brought new hope to the treatment of diabetic patients. In particular, the lateral differentiation ability of adult stem cells (that is, the differentiation of adult stem cells into specific cells and tissues under the action of a certain microenvironment) provides an alternative source of cells for cell transplantation for diabetes. Mesenchymal stem cells (MSC) are the most representative adult stem cells, which not only have the potential to induce differentiation into insulin-secreting cells, but also grow rapidly *in vitro*, and also have special low immunity (expressing low levels of major histocompatibility complex II). Strong immune regulation and tissue repair capabilities have attracted much attention. It has been known that MSC can play a role in the development of diabetes. Therefore, in recent years, MSC treatment of diabetes has become a research hotspot. MSCs are widely available, and the most widely studied are MSCs derived from bone marrow and umbilical cord. For example, bone marrow-derived MSC (BMSC) is used to treat newly diagnosed type 1 diabetes patients. No acute or chronic side effects occur during use. After 12 months of follow-up, the patient's insulin requirement is reduced by 30-60%, and serum C-peptide levels are elevated. 2-10 times. Moreover, Badri et al. conducted a META analysis of 12 independent clinical study, and found that the efficacy of umbilical cord mesenchymal stem cells in the treatment of T1DM was not significant.

Although the use of these sources of MSC for the treatment of diabetes has a certain clinical effect, there are several potential problems: 1) low yield, slow production, limited use of large-scale expansion *in vitro*; 2) MSC obtained from bone marrow has certain clinical traumatic; 3) long-term use of bone marrow MSC therapy has potential tumorigenic risk; 4) because umbilical cord and bone marrow MSC are mostly allogeneic, hematopoietic stem cells cannot be completely excluded, and mixed allogeneic hematopoietic stem cell transplantation can induce fatal concurrency in recipients. Symptoms, such as acute graft-versus-host response (aGVHD), significantly increase the risk of patient care. Once a GVHD has occurred, the mortality rate is as high as 52%.

Recent studies have found that gingival mesenchymal stem cells (GMSC) not only have

the characteristics of general MSC, but also easy to obtain separation, uniform culture *in vitro*, rapid proliferation, no growth factor, no serum, no tumorigenic, stable phenotype and telomerase activity, and has unique advantages such as autologous source and ethical controversy, making it an ideal source of MSC for clinical treatment. Our group first reported that GMSC can treat autoimmune arthritis and has applied for GMSC treatment of various autoimmune diseases (AID). In addition, using animal humanization models, we analyzed that GMSCs inhibit human cell initiation and that GMSCs are better than BMSCs, which is capable of treating T1DM mice in a diabetic mouse model. Importantly, we have completed the GLP test in line with national standards in the early stage and found that GMSC is highly safe. This project is based on extensive preliminary researches having an internationally advanced level of previous work. It observes the clinical treatment research of GMSC in patients with type 1 diabetes, explores the clinical treatment plan that can be promoted, has broad application prospects and has great society and economic benefits.

2. Study objectives

The objectives of this trial includes: 1. To evaluate the efficacy of GMSCs in the treatment of type 1 diabetes; 2. To evaluate the safety of GMSCs therapy for type 1 diabetes; 3. To explore the regulatory function and possible mechanisms of gingival stromal cells in patients with type 1 diabetes.

3. Trial Design

The study is a prospective, monocenter, randomized, double-blind, placebo-controlled clinical trial that evaluates the safety and efficacy of gingival mesenchymal stem cells in the treatment of type 1 diabetes. The study is registered (chictr.org.cn, 1800018339).

After screening by inclusion criteria and exclusion criteria, the subjects were divided into the stem cell trial group and the placebo-controlled group according to the principle of randomized and parallel controlled trial design. The placebo-controlled will be given routine insulin therapy + placebo infusion; the stem cell trial group will be given routine insulin therapy + GMSCs infusion.

Routine insulin therapy: multiple subcutaneous injections of insulin or insulin pump therapy to achieve blood glucose control. The standard of blood glucose control is as follows: fasting fingertip blood glucose (FBG) is 4.4mmol/L-5.6mmol/L, and postprandial fingertip blood glucose (PBG) is <7.8 mmol/L.

GMSCs infusion: 200mL GMSCs mixture containing 1.0×10^6 / Kg body weight GMSCs, a total of 4 infusions, once every 3 weeks for 9 weeks.

Placebo infusion: 200mL saline, a total of 4 infusions, once every 3 weeks for 9 weeks.

Trial period: Treatment period of 9 weeks (visit 1 to 4 at week 0, 3, 6, 9), follow-up period of 39 weeks (visit 5 to 9 at week 12, 18, 24, 36, 48).

3.1. Inclusion criteria:

a. Aged from 18 to 60 years old without sex limitation.

b. Signed informed consent form;

c. Mentally normal and able to comply with the procedures of the trial protocol;

d. Meets the WHO 1999 diagnostic criteria for type 1 diabetes mellitus (T1DM);
e. Duration of T1DM ≤ 10 years at enrollment;
f. Baseline C-peptide level ≤ 0.3 ng/mL;
g. Women with childbearing potential must have no pregnancy plans within 1 year.

3.2. Exclusion criteria:

If any of the following criteria are met, the subject will not be allowed to enter this study:
a. Body mass index (BMI) >30 kg/m²;
b. Daily insulin requirement >100 U/day;
c. Aspartate aminotransferase (AST) or alanine aminotransferase (ALT) >2.5 times the upper limit of normal and/or serum creatinine (Cr) ≥133 μmol/L (1.5 mg/dL);
d. Severe hepatic or renal dysfunction, proliferative diabetic retinopathy, or macular edema;
e. History of coronary angioplasty, coronary stent implantation, coronary artery bypass grafting, myocardial infarction, unstable angina, clinically significant ECG abnormalities, cerebrovascular accident, or transient ischemic attack;
f. Acute or chronic pancreatitis; active infections (including hepatitis C, HIV, or tuberculosis confirmed by positive skin test or clinical manifestations), or suspected tuberculosis requiring treatments;
g. Long-term systemic corticosteroid therapy; bleeding disorders (e.g. hemophilia, idiopathic thrombocytopenic purpura);
h. Malignancy, psychiatric disorders, or hypersensitivity to mesenchymal stem cells (MSC) or preservation fluid;
i. Pregnant or breastfeeding women;
j. Other severe comorbidities deemed unsuitable for inclusion by the investigator.

3.3. Randomization and blinding

Trial grouping: The block randomization method is used, and the SPSS 24.0 statistical software is used to generate a random number grouping table according to the random proportion.

Implementation method: Random grouping is used, and subjects have a 50% chance of entering the stem cell trial group or the control group.

Blinding: The group allocation results are known only to third-party personnel. Both patients and clinical investigators remain blinded to the group assignments until the end of the trail. During each treatment, both groups will receive an intravenous administration following the same protocol, and solution will be packaged in a brown opaque bag to prevent differentiation by appearance. Once the database has been locked, and populations for analysis agreed, all codes (subject identification and randomization) will be re-checked.

3.4. Sample size

This Phase I clinical trial employs a fixed sample size design, with 20 participants planned for enrollment per group. The final analysis will be based on the actual number of recruited subjects.

4. Variables

4.1. Efficacy variables

4.1.1. Primary endpoint

The primary endpoint is the change of β-cell function from baseline assessed at week 12 (the first visit after 4 infusions). Subjects are provided a mixed-meal, and C-peptide levels are observed within 2 hours after food intake (0, 30, 60 and 120 minutes). The fasting C-peptide, peak C-peptide and the area under the curve (AUC) of C-peptide calculated by formula are used to assess the β-cell function.

4.1.2. Secondary endpoints

a) Change of β-cell function from baseline assessed at week 18, 24, 36, 48 (the scheduled visits during follow-up period). Subjects are provided a mixed-meal, and C-peptide levels are observed within 2 hours after food intake (0, 30, 60 and 120 minutes). The fasting C-peptide, peak C-peptide and the AUC of C-peptide calculated by formula are used to assess the β-cell function;

b) Exogenous insulin demanded quantity change assessed at week 3, 6, 9, 12, 18, 24, 36, 48 (the scheduled visits throughout the trial);

c) Glycometabolism index assessed at scheduled visits throughout the trial:

- Hemoglobin A1c (HbA1c) levels at week 12, 24, 36, 48 (the scheduled visits every 12 weeks throughout the trial);
- Fasting blood glucose (FBG) assessed at week 3, 6, 9, 12, 18, 24, 36, 48 (the scheduled visits throughout the trial);
- 2h-postprandial blood glucose (PBG) assessed at week 3, 6, 9, 12, 18, 24, 36, 48 (the scheduled visits throughout the trial);
- The AUC of blood glucose at week 12, 18, 24, 36, 48 (the scheduled visits during follow-up period). Subjects are provided a mixed-meal, and blood glucose levels are observed within 2 hours after food intake (0, 30, 60 and 120 minutes). The AUC of blood glucose are calculated by formula.
- Time in range (TIR) assessed at week 3, 6, 9, 12, 18, 24, 36, 48 (the scheduled visits throughout the trial). TIR measures the percentage of time a person's glucose levels stay within target range (3.9-10.0 mmol/L).
- Standard deviation of blood glucose (SDBG) assessed at week 3, 6, 9, 12, 18, 24, 36, 48 (the scheduled visits throughout the trial). SDBG quantifies glucose variability by calculating the standard deviation of blood glucose over a day.
- Coefficient of variation (CV) assessed at week 3, 6, 9, 12, 18, 24, 36, 48 (the scheduled visits throughout the trial). CV measures relative glucose variability by calculating the percentage ratio of standard deviation (SD) to mean blood glucose.
- Largest amplitude of glucose excursion (LAGE) assessed at week 3, 6, 9, 12, 18, 24, 36, 48 (the scheduled visits throughout the trial). LAGE quantifies the single most extreme glucose swing within 24 hours by calculating the difference between the highest and lowest blood glucose.

4.1.3. Exploratory endpoints

The exploratory endpoints include immunological and inflammatory indicators, oxidative stress markers, diabetic complication evaluation. Exploratory analyses of these endpoints will be performed using collected biological samples (including plasma, urine and isolated peripheral blood mononuclear cells) and on a case-specific basis. The final exploratory analyses will be determined based on actual situation.

4.2. Safety variables

Patient safety will be assessed through the collection of reported adverse events (AEs) and clinical laboratory data.

4.2.1. Adverse events (AEs)

Any adverse event that occurs to a subject during the course of the trial (from the date of signing an informed consent form to the last follow-up) is defined as an adverse event (AE), regardless of whether it is related to the treatment.

A serious adverse event (SAE) is one of the following medical events that occurs during the clinical trial using any dose of the test drug: leading to death or life-threatening (here "life-threatening" means that the patient is at risk of death when a serious adverse event occurs, rather than assuming that a serious adverse event may cause death if it is more serious); requiring hospitalization or prolonged hospitalization; causing permanent or severe disability/loss of function; congenital malformation or birth defect; an event of important medical significance.

Adverse events of special interest (AESI) are AEs (serious or non-serious) required to be monitored, documented, and managed in a pre-specified manner. The AESI of this trial is hypoglycemia event, which will be documented using dedicated tick boxes when a subject suffered from any of the following situation:

- Severe hypoglycemia: events requiring active administration of carbohydrates, glucagon, or other resuscitative actions by another person. Blood glucose confirmation is not mandatory.
- Symptomatic hypoglycemia: there are typical symptoms of hypoglycemia, and blood glucose $\leq$ 3.9 mmol/L.
- Suspected symptomatic hypoglycemia: there are symptoms of hypoglycemia, but no blood glucose measurement has been performed.
- Asymptomatic hypoglycemia: there are not typical symptoms of hypoglycemia, but blood glucose $\leq$ 3.9 mmol/L.
- Pseudohypoglycemia: there are symptoms of hypoglycemia, but blood glucose $>$ 3.9mmol/L.

Severe hypoglycemia, symptomatic hypoglycemia, suspected symptomatic hypoglycemia and asymptomatic hypoglycemia will be enrolled in hypoglycemia adverse event analysis, while pseudohypoglycemia will be excluded.

4.2.2. Laboratory data related to safety evaluation

a) Hepatic and renal function assessed at week 3, 6, 9, 12, 18, 24, 36, 48 (the scheduled visits throughout the trial), including alanine aminotransferase (ALT), aspartate aminotransferase (AST), creatinine, blood urea nitrogen (BUN), uric acid (UA).
b) Blood lipids assessed at week 12, 24, 36, 48 (the scheduled visits every 12 weeks throughout the trial), including total cholesterol, triglyceride, low-density lipoprotein cholesterol and high-density lipoprotein cholesterol.

5. Statistical Consideration

5.1. Timing of analyses

The final analysis will be performed after the last subject has completed the final visit at week 48 and the database has been locked. The randomization code will not be broken before the finalization, approval and publication of this SAP document.

5.2. Study population

5.2.1. Efficacy population

The analysis of efficacy will be performed in the full analysis population (all participants who have undergone randomization) according to the intention-to-treat principle. The intention-to-treat principle will be the main strategy of analysis adopted for primary and secondary endpoints.

5.2.2. Safety population

The analysis of safety will be performed in all randomized subjects who received at least 1 treatment in the intention-to-treat population. All subjects who received any treatment (including control) but excluding subjects who drop out prior to receiving any treatment are included.

5.3. Missing data

This section includes the methods for missing data imputation for some analyses, if necessary.

5.3.1. Missing data for primary endpoints

For missing data of the primary endpoints, last observation carried forward (LOCF) will be used for imputation under the assumption that data are missing at random (MAR). This approach is justified given: a) chronic stable disease state for type 1 diabetes; b) short study visiting interval (3 weeks).

5.3.2. Missing data for secondary endpoints

Missing data will not be imputed.

5.3.3. Missing data for date and time of treatment and visiting

Since the treatment and visiting is administered at the hospital, the date and time of study administration are filled in e-CRF. No missing data is expected.

5.4. The general statistical principles

All statistical analyses were performed by using R package 'stats' in R language (ver 3.4.3 or the latest version). The *P* value was two-sided for all end points and a *P* value of less than 0.05 was considered to indicate statistical significance.

If the data within GMSCs group and placebo group under comparison satisfy the assumptions of normality (assessed using the Shapiro-Wilk test, significance level α = 0.05 for testing, combined with visual inspection of quantile-quantile plots and histograms) AND homogeneity of variances (assessed using Levene's test, significance level α= 0.1), the independent samples Student's t-test will be employed to evaluate the statistical significance of between-group differences in means.

If the data within any group under comparison violate the assumption of normality OR violate the assumption of homogeneity of variances, the non-parametric Wilcoxon rank-sum test will be utilized to evaluate the statistical significance of between-group differences in distributions.

The primary analysis of the primary endpoints will be unadjusted. All other statistical analyses will be unadjusted.

5.5. Demographics and baseline characteristics

For each patient, demographics and baseline characteristics will be obtained from the last available value up to the date of the first study treatment administration (i.e. baseline definition). For patients randomized and not treated, the baseline value is defined as the last available measurement prior to the date of randomization. The following variables will be summarized:

Demographics

(1) Sex (Male, Female).

(2) Age in years.

(3) Baseline body weight (kg).

(4) Baseline body mass index (BMI) in kg/m².

(5) The duration of T1DM in years.

Baseline characteristics

(1) Indexes of glucose metabolism: fasting blood glucose (mmol/L), 2wo-hour postprandial blood glucose (mmol/L), AUC of blood glucose during a 2-hour mixed-meal tolerance test and calculated using the trapezoidal rule (mmol/L*h).

(2) Indexes of β-cell function: fasting C-peptide (ng/ml), peak C-peptide (ng/ml), AUC of C-peptide during a 2-hour mixed-meal tolerance test and calculated using the trapezoidal rule (ng/ml*h).

(3) Daily insulin dose (IU/d/kg).

(4) Hemoglobin A1c level (%) and glycated Albumin (%).

(5) Antibodies related to T1DM: islet cell antibody (COI), insulin autoimmune antibodies (COI), glutamic acid decarboxylase antibody (IU/ml), insulinoma associated-2 antibody (IU/ml).

(6) Hepatic and renal function: alanine aminotransferase (U/L), aspartate aminotransferase (U/L), creatinine (μmol/L), blood urea nitrogen (mmol/L), 24-hour urine albumin (g), urinary albumin to creatinine ratio (mg/g), uric acid (μmol/L).

(7) Blood lipids: total cholesterol (mmol/L), triglyceride (mmol/L), low-density lipoprotein cholesterol (mmol/L), high-density lipoprotein cholesterol (mmol/L).

Demographics and other baseline characteristics will be analyzed using descriptive statistics. Continuous variables will be summarized using mean, standard deviation, median, interquartile range, maximum, and minimum values. Categorical variables will be summarized using count (n) and percentage (%).

5.6. Analysis of primary endpoints

For β-cell function, the AUC of C-peptide will be assessed during a 2-hour mixed-meal tolerance test and calculated using the trapezoidal rule. The change from baseline of the AUC of C-peptide (ng/ml*h), peak C-peptide (ng/ml) and the fasting C-peptide (ng/ml) at week 12 will be compared between the two groups, with the statistical methodology adhering to the general statistical principles mentioned above.

5.7. Analysis of secondary endpoints

For β-cell function, the AUC of C-peptide will be assessed during a 2-hour mixed-meal tolerance test and calculated using the trapezoidal rule. The change from baseline of the AUC of C-peptide (ng/ml*h), peak C-peptide (ng/ml) and the fasting C-peptide (ng/ml) at week 18, 24, 36, 48 will be compared between the two groups, with the statistical methodology adhering to the general statistical principles mentioned above..

For daily insulin doses, the fold change relative to baseline at week 3, 6, 9, 12, 18, 24, 36, 48 will be compared between the two groups, with the statistical methodology adhering to the general statistical principles mentioned above.

For glucose metabolism indicators, the levels of HbA1c, FBG, PBG, AUC of blood glucose, TIR, SDBG, CV and LAGE at their respective scheduled assessment time points (see section 4.1.2) will be compared between the two groups, with the statistical methodology adhering to the general statistical principles mentioned above.

5.8. Analysis of exploratory endpoints:

These may include single-cell RNA sequencing, flow cytometry, omics profiling and other advanced analyses. The final exploratory analyses will be determined based on actual situation.

5.9. Analysis of safety data

5.9.1. Analysis of adverse events

All AEs (including serious adverse events (SAE) and AEs of special interest) diagnosed and recorded by the investigator will be reported and described. And all safety analyses will be stratified by treatment group.

The severity of the AEs is graded as follows:

- Mild: symptoms appear but not affect normal life. And no symptomatic treatment or drug withdrawal is required.
- Moderate: symptoms appear and have mild effects on normal life. And no symptomatic

treatment or drug withdrawal is required.

- Severe: symptoms appear and affect normal life, or symptomatic treatment or drug withdrawal is required.
- Life-threatening: symptoms are severe, life-threatening, fatal or disabling, and the drug needs to be stopped immediately or emergency treatment is required.
- Death: subject die.

The five principles for evaluating the association between drugs and adverse events are:

① Is there a reasonable temporal relationship between the use of the drug and the occurrence of the adverse event?

② Does the event match the known adverse event types of the drug?

③ After discontinuing or reducing the dosage of the drug, does the event disappear or alleviate?

④ When the suspected drug is used again, does the same event reappear?

⑤ Can the event be explained by the effects of concurrent medications, the progression of the patient's condition, or the impact of other treatments?

Correlation evaluation: Based on the above five principles, the association is divided into six levels: definitely relevant, probably relevant, might be relevant, probably irrelevant, to be evaluated and unable to be evaluated.

Judgment indicators / Judgment results	1	2	3	4	5
It is definitely relevant	+	+	+	+	-
It is probably relevant	+	+	+	?	-
It might be relevant	+	±	±?	?	±?
It probably irrelevant	-	-	±?	?	±?
To be evaluated	Additional information is needed for evaluation				
Unable to be evaluated	The necessary information for evaluation could not be obtained				

Note: + means yes; - means no; ± means difficult to be sure or deny; ? means unknown.

The AEs will be summarized in: person-time (defined as the total count of AEs

occurrences across all subjects during the observation period), number of subjects (n) and percentage (%) in each treatment group.

5.9.2. Analysis of laboratory data

Laboratory data pertaining to safety evaluation will be compared between the two groups at week 12 and 48 (at the first visit after 4 infusions and the end of the trial), with the statistical methodology adhering to the general statistical principles mentioned above.
