# Supplementary material for: Safety and efficacy of human gingiva-derived mesenchymal stem cells in patients with type 1 diabetes: a double-blind, randomized, placebo-controlled, phase 1 trial
Source: Signal Transduct Target Ther. 2026 Sep 30;11:434. doi: 10.1038/s41392-026-03021-8 (PMC13627707; doi:10.1038/s41392-026-03021-8)
Supplement: Supplementary file 2 [file 41392_2026_3021_MOESM3_ESM.pdf]

Version number: SYSUZSG17414-2.0
Version date: January 23, 2022

Clinical Trial protocol of human gingival mesenchymal stem cell therapy for type 1 diabetes mellitus

Study Name: A Clinical Trial for Human Gingival Mesenchymal Stem Cells in the Treatment of Type 1 Diabetes Mellitus
Investigational product under study: Human gingival mesenchymal stem cell injection
Program Coordination Meeting Date: January 6, 2018
Date of finalization: March 12, 2018
Date of revision: January 23, 2022

Clinical trial participant Institution: The Third Affiliated Hospital of Sun Yat-sen University
Principal investigator: Professor Yanming Chen / Song Guo Zheng

Data management and statistical analysis: School of Public Health, Sun Yat-sen University
Statistical Leader: Professor Ling Li

Sponsor: The Third Affiliated Hospital of Sun Yat-sen University
Person in charge: Professor Yanming Chen
Contact number: 18922102818
Address of the Sponsor: NO.600 Tianhe Road, Tianhe District, Guangzhou, Guagndong, China
Zip Code: 510630

==

Confidentiality Statement

This trial protocol is confidential and is provided to medical experts, researchers, and other personnel involved in the trial, as well as relevant institutions such as healthcare institutions, ethics committees, and contract research organizations undertaking the trial. Without prior written consent from the sponsor, the content of this protocol shall not be disclosed or leaked to any third part. Furthermore, when part or all of the results of this clinical trial are published to external societies,journals,etc.,the written consent of the sponsor is required.

Program·Signature Page

Investigational product under study: Gingival mesenchymal stem cell injection
Study Name: A Clinical Trial for Human Gingival Mesenchymal Stem Cells in the treatment of Type 1 Diabetes Mellitus
Clinical Trial protocol version/Version date: version 2.0 / January 23, 2022

1. Declaration of the Sponsor

In accordance with the "Measures for the Administration of Stem Cell Clinical Research (Trial)", I will be responsible for initiating, applying for, organizing, funding and monitoring this clinical research, especially providing financial compensation for treatment-related damages or deaths, and providing legal and financial guarantees to the researchers.

Sponsor: The Third Affiliated Hospital of Sun Yat-sen University
Project leader (signature): _______ Date: __Year __ month__day

2. Declaration of Statisticians

In accordance with the "Measures for the Administration of Stem Cell Clinical Research (Trial)", I will be responsible for the data management and statistical analysis of this clinical trial, conscientiously perform the duties of a statistician, and ensure the authenticity, science and standardization of statistical results.

Statistical expert Institution ： _________________
Statistical director (signature): _______ Date: __ Year __ month__day

3. Declaration of Investigators

I have read the protocol carefully and agree to carry out this clinical trial according to the protocol.

Clinical trial participants
Principal investigator (signature): _______ Date: __ Year __ month__day

Abbreviations

Abbreviations	Full name
AID	Autoimmune Disease
T1DM	Type 1 diabetes mellitus
MSCs	Mesenchymal stem cells
GMSCs	Gingival mesenchymal stem cells
RBC	Red blood cell
HB	Hemoglobin
WBC	White blood cell
FBG	Fasting blood glucose
PBG	Blood glucose after meal
NEUT	Proportion of neutrophils
8-OH-dG	8-Oxo-2'-deoxyguanosine
PLT	Platelet
CRF	Case report form
FAS	Full analysis set
PPS	Per Protocol Set
SAS	Safety Analysis Set
SOP	Standard Operating Procedure
ADR	Adverse Drug Reaction
AE	Adverse event
SAE	Serious adverse events
ALT	Alanine transaminase
AST	Aspartate transaminase
Cr	Creatinine
HbA1c	Glycosylated Hemoglobin, Type A1C
GAD65	Glutamic acid decarboxylase 65-kilodalton isoform
IA2	Islet antigen 2

Clinical trial protocol for treatment of type 1 diabetes with human gingival mesenchymal stem cells

1. Study Name : A Clinical Trial for Human Gingival Mesenchymal Stem Cells in the treatment of Type 1 Diabetes Mellitus

2. Research objectives

1) To evaluate the efficacy of gingival mesenchymal stem cells (GMSCs) therapy for type 1 diabetes;

2) To evaluate the safety of GMSCs therapy for type 1 diabetes;

3) To explore the regulatory function and potential mechanism of GMSCs in patients with type 1 diabetes.

3. The Basis of the Topic

The cost of diabetes treatment is staggering, accounting for 12% of global health expenditure. The latest data indicates that the prevalence of adult diabetes in China has reached 11.0%, with a total of 113.9 million patients. Among them, 7%-12% have type 1 diabetes (T1DM) , and the incident rate is increasing by 3% annually. T1DM is an autoimmune disease mediated by T cells that occurs in genetically susceptible individuals, in which the function and/or capacity of pancreatic β cells are defective and reduced, leading to insufficient insulin secretion to meet the body's needs. Currently, the primary treatment relies on insulin replacement therapy. However, this approach cannot accurately mimic the regulatory role of pancreatic β-cells in glucose homeostasis, and as the disease progresses, it can lead to various complications with high mortality and disability rate. Exploring methods to improve or repair pancreatic β-cell damage is crucial for advancing the field of diabetes treatment.

Maintaining or reconstructing pancreatic function through various methods has been a key focus in diabetes treatment. Despite significant progress in establishing endogenous insulin secretion systems through methods like pancreatic or islet transplantation, the severe shortage of donor organs, low survival rates of islet cells *in vitro*, and post-transplant immune rejection have severely limited their clinical application. In recent years, the rapid development of stem cell theory and technology has brought new hope to the treatment of diabetes patients. Particularly, the confirmation of the lateral differentiation ability of adult stem cells (i.e., the ability of adult stem cells to differentiate into specific cells and tissues under certain microenvironmental conditions) has provided alternative cell source for cell transplantation therapy in diabetes.

Mesenchymal stem cells (MSCs) are the most representative adult stem cells, capable of differentiating into insulin-secreting cells. They grow rapidly *in vitro* and exhibit low immunogenicity (expressing low levels of MHC-II), strong immune regulation, and tissue repair capabilities, which have made them a focus of research. It is now known that MSCs play a role in multiple stages of diabetes development, making MSC therapy a recent research hotspot. MSCs can be derived from a wide range of sources, with bone marrow and umbilical cord being the most extensively studied. For example, bone marrow-derived mesenchymal stem cells (BMSCs) have been used to treat newly diagnosed T1DM patients without causing acute or chronic side effects. After a 12-month follow-up, the patients' insulin requirements

decreased by 30-60%, and serum C-peptide levels increased by 2-10 times. However, Badri et al. conducted a META analysis of 12 independent clinical studies and found that the efficacy of umbilical cord mesenchymal stem cell therapy in treating T1DM was not significant. While using these MSCs to treat diabetes has shown some clinical benefits, several potential issues remain: 1) low yield, slow manufacturing processes, and limitations in large-scale *in vitro* expansion; 2) obtaining MSCs from bone marrow can be clinically invasive; 3) long-term use of BMSCs for treatment carries a risk of tumorigenesis; 4) since most MSCs from umbilical cords and bone marrow are allogeneic, it is impossible to completely rule out hematopoietic stem cells and mixed allogeneic hematopoietic stem cell transplantation can induce fatal complications in recipients, such as acute graft-versus-host disease (aGVHD), significantly increasing the risk of treatment. The mortality rate for aGVHD can be as high as 52%. Recent studies have shown that gingival mesenchymal stem cells (GMSCs) not only possess the typical characteristics of MSCs, but also offer several unique advantages. They are easy to obtain and isolate, can be cultured uniformly *in vitro*, proliferate rapidly, do not require growth factors or serum, are not tumorigenic, maintain stable phenotypes and telomerase activity even after long-term culture, and can be autologous without ethical concerns. These features make GMSCs an ideal source of MSCs for clinical treatment.

Our research team has first reported that GMSCs can treat autoimmune diseases (AID) and applied a patent for GMSCs treatment of AID. Additionally, using animal models, we have analyzed that GMSCs can inhibit the onset of human cell-mediated diseases, and their effectiveness surpasses that of BMSCs. In a diabetic mouse model, GMSCs can effectively treat T1DM mice. Importantly, we have completed GLP trials that meet national standards, confirming the safety of GMSCs.

This project is an original research at the international leading level based on a large number of preliminary work. It observes the clinical treatment of GMSCs in patients with T1DM, explores the clinical treatment scheme that can be popularized, and has broad application prospects and great social and economic benefits.

4. Expected Effects

The treatment of GMSCs is safe and effective for T1DM. It can improve the pancreatic function of patients with T1DM and alleviate the symptoms of diabetes.

5. Research Design

5.1 The Design of Clinical Research Protocol Is in Accordance With Ethical Requirements

1. This clinical study protocol complies with the relevant provisions of the Declaration of Helsinki on the protection of subjects' rights and interests.
2. The clinical study protocol is agreed upon by the principal investigator and sponsor, and is approved by the ethics committee before the trial begins.
3. The opinion of the ethics committee may be to approve, to approve with necessary amendments, to disapprove, to terminate or to suspend an approved trial.
4. If problems occur in the process of clinical research and the protocol needs to be modified, the revised protocol shall be approved by the ethics committee before implementation.

5. If important new information involving investigational biological products is found, the informed consent form must be modified in writing and submitted to the ethics committee for approval, and the consent of the subject must be obtained again.

5.1.1 Protection of the privacy of subjects

Only researchers and monitors involved in clinical studies have access to the personal medical records of participants, which will be kept confidential. The drug supervision and administration department and the national health supervision department have the right to inspect clinical research records. Data processing will be conducted anonymously, omitting any personal information that could identify participants. The personal medical information of participants will be stored at the National Drug Clinical Research Base or the hospital research department.

5.1.2 Informed consent process

Physicians participating in clinical research must provide subjects with detailed information about the study, including its purpose, nature, potential benefits and risks, alternative treatments, and the rights and obligations of subjects as outlined in the Declaration of Helsinki, so that the subjects can understand and agree. Only after voluntarily signing the informed consent form can the clinical research be proceeded. If significant new information related to the investigational products are discovered during the clinical study, the informed consent form must be revised in writing, re-approved by the ethics committee, and the subjects' consent must be obtained again.

5.2 Study Protocol Design

1) Trial design

The study is a prospective, monocenter, randomized, double-blind, placebo-controlled clinical trial that evaluates the efficacy and safety of GMSCs in the treatment of type 1 diabetes.

2) Trial grouping

This study employs a block randomization method. The random numbers are generated by statistical professionals using SPSS 24.0 software on a computer. All random numbers are distributed to the researchers, and they select the numbers in ascending order based on the sequence of each participant's enrollment. Number skipping is strictly prohibited to ensure that participants are randomly assigned to either the stem cell treatment group or the placebo-control group.

3) Control drug

Placebo control will be used in this trial. All patients have insulin as the basic treatment, and during the trial period, the patient's insulin dose should be as stable as possible.

6. Study Criteria and Remedial Measure

6.1 Inclusion criteria:

a. Aged from 18 to 60 years old without sex limitation.

b. Signed informed consent form;

c. Mentally normal and able to comply with the procedures of the trial protocol;

d. Meets the WHO 1999 diagnostic criteria for type 1 diabetes mellitus (T1DM);

e. Duration of T1DM ≤ 10 years at enrollment;

f. Baseline C-peptide level ≤ 0.3 ng/mL;

g. Women with childbearing potential must have no pregnancy plans within 1 year.

6.2 Exclusion criteria:

If any of the following criteria are met, the subject will not be allowed to enter this study:

a. Body mass index (BMI) >30 kg/m²;

b. Daily insulin requirement >100 U/day;

c. Aspartate aminotransferase (AST) or alanine aminotransferase (ALT) >2.5 times the upper limit of normal and/or serum creatinine (Cr) ≥133 µmol/L (1.5 mg/dL);

d. Severe hepatic or renal dysfunction, proliferative diabetic retinopathy, or macular edema;

e. History of coronary angioplasty, coronary stent implantation, coronary artery bypass grafting, myocardial infarction, unstable angina, clinically significant ECG abnormalities, cerebrovascular accident, or transient ischemic attack;

f. Acute or chronic pancreatitis; active infections (including hepatitis C, HIV, or tuberculosis confirmed by positive skin test or clinical manifestations), or suspected tuberculosis requiring treatments;

g. Long-term systemic corticosteroid therapy; bleeding disorders (e.g. hemophilia, idiopathic thrombocytopenic purpura);

h. Malignancy, psychiatric disorders, or hypersensitivity to mesenchymal stem cells (MSC) or preservation fluid;

i. Pregnant or breastfeeding women;

j. Other severe comorbidities deemed unsuitable for inclusion by the investigator.

6.3 Exit criteria

1. The patient has an allergic reaction or other serious adverse event when using the requirements of this trial, and the investigator determines that continuing the study will endanger the safety of the subject;

2. The subject himself requests to withdraw;

3. Unplanned pregnancy;

4. Remedial medication after 12 weeks, HbA1c>9% or an increase of more than 1% over baseline.

6.4 Cases of drop-out

1.Determination of case drop-out: Subjects who do not complete the whole observation of this study are all dropped out cases.

2.Treatment of case drop-out: Researchers should do everything possible to keep subjects in the study for as long as possible. When a drop-out case occurs, researchers must record the reason for the drop-out in the CRF form, and try to contact the patient to complete the assessment items that can be completed, and try to fill in the treatment non-follow-up record form to record the last time of taking medication.

Participants who withdrew from the study cannot be re- enrolled in this study. Their selection and treatment numbers cannot be reused.

6.5 Remedial measure

For rescue treatment, use rapid-acting insulin for intensive therapy. Administer the maximum dose of the drug used in this study, and at follow-up, check that the HbA1c level is greater than 9%, or has increased by more than 1% from baseline.

After CSII intensive treatment for 2 weeks, the blood sugar level reached the standard, and then continued to be implemented according to the group. If the HbA1c level remains unmet 3 months after implementing rescue measures, the treatment will be considered ineffective, and the participant will be withdrawn from the study, but their data will still be included in the final analysis.

Remedial measures should be implemented within one month of receiving the results of a centrally tested HbA1c.

7. Number of Cases Required

This Phase I clinical trial employs a fixed sample size design. The stem cell trial group and the placebo-control group are in a ratio of 1:1, with 20 cases in the stem cell trial group and 20 cases in the placebo-control group, focusing on the efficacy and safety of GMSCs treatment.

8. Stem Cell Preparation

8.1 GMSCs isolation, culture and injection preparations

Healthy human gingival tissues (from healthy Asian male or female during wisdom tooth extraction surgery, aged between 20 and 30 years old, without any autoimmune or inflammatory diseases) will be obtained as remnant or discarded tissues at the Third Affiliated Hospital of Sun Yat-sen University, following institutional review board (IRB) approval and informed consent. Stem cell isolation, expansion, quality control, and cryopreservation will be all conducted under strict laboratory conditions in compliance with current Good Manufacturing Practice of Medical Products (GMP) standards in Zhongshan Ophthalmic Center of Sun Yat-sen University.

8.2 Trial Grouping and Dosing Regimen

Stem cell trial group: routine insulin treatment + GMSCs.

Placebo-control group: routine insulin treatment + placebo infusion;

Routine multiple subcutaneous injections insulin or insulin pump therapy should be used to achieve the standard of blood glucose control: FBG range 4.4mmol/L-5.6mmol/L and PBG <7.8 mmol/L.

Usage, dosage, timing, and duration of stem cell preparations: Infuse 200mL of GMSCs injection (containing 1.0×10^6 stem cells/kg body weight) over 9 weeks, with 4 infusions every 3 weeks. Before GMSCs infusion, adjust insulin to minimize blood glucose fluctuations in patients.

Cell infusion method: Based on our preclinical studies, GMSCs are infused into the tail vein of mice with T1DM. This treatment can effectively alleviate inflammation and treat T1DM. Currently, there are no international clinical studies on using GMSCs to treat diabetes. However, based on the clinical trials of other mesenchymal stem cells, all treatments have been administered via intravenous injection. Considering both our preclinical research and the clinical trial experiences of other researchers, we have adopted the same injection method.

Trial period: Treatment period of 9 weeks (visit 1 to 4 at week 0, 3, 6, 9), follow-up period of 39 weeks (visit 5 to 9 at week 12, 18, 24, 36, 48).

Precautions: Before injection, the solution should be left at room temperature for 40-50 minutes to allow it to warm up. Shake the solution gently before injection to ensure thorough mixing of the cells; administer the infusion slowly over about 1 hour. Monitor vital signs closely during the infusion process. After each treatment session, patients should remain in the hospital for 30 minutes of observation and one week of outpatient follow-up.

8.3 Packaging, Management and Transportation of GMSCs

8.3.1 Packaging

All the GMSCs injection solutions used in the trial have corresponding preparation numbers and are labeled. Both the test drug and the control drug are uniformly stored in light-protective infusion bags. Each participant receives only one injection per session. Each light-protective bag must be labeled with the participant's number, the preparation name, its indications, specifications, usage instructions, storage conditions, the preparation supplier, and the phrase "for clinical research use only."

8.3.2 Formulation Management and Transportation

1) According to the regulations of GCP on the management of experimental drugs, the external label of the experimental injection shall be unified and marked as special for clinical trials.

2) According to the agreed treatment time between the investigator and the subject, the sponsor will deliver the trial injection to the trial center before the start of treatment. The sponsor is responsible for the transportation of the trial injection.

3) The transportation of the injection should comply with relevant requirements and regulations, monitoring parameters such as temperature (2°C-8°C) and airtightness during transportation. The transportation time of the cell preparation should be shortened as much as possible to ensure that it is delivered to the clinical trial research hospital within two hours. Additionally, a backup transportation plan is required to prevent emergencies or unexpected situations that may arise during the transportation of the injection.

4) Each time the test injection solution is used, the time from preparation to administration must be controlled within 8 hours. Unless in special circumstances, if the subject does not appear at the agreed time and is more than 2 hours late, the research institution must set up a dedicated location for storage, maintaining the temperature between 2°C and 8°C. However, it must be ensured that the administration is completed within the 8-hour validity period of the preparation. If the validity period is exceeded, the solution must be discarded, and the sponsor must be promptly notified for disposal.

9. Criteria for suspension and termination of clinical studies

9.1 Criteria for Suspension of Clinical Studies

Trial suspension means that the clinical research has not been completed according to the plan, and the entire trial is stopped midway. The purpose of trial suspension is mainly to protect the rights of subjects, ensure the quality of the trial and avoid unnecessary economic losses.

1) If serious safety problems occur in the trial, the trial should be stopped in time;

2) In the trial, it is found that there are major errors in the clinical research scheme, which makes it difficult to evaluate the effect of the trial product; or a well-designed scheme has serious deviations in the implementation, and it is difficult to evaluate the effect of the product if it continues;

3) The sponsor requests to suspend the application due to financial, management and other reasons;

4) The State Food and Drug Administration orders the trial to be suspended for some reason.

9.2 Criteria for Termination of Clinical Studies

1) Serious adverse events occur, and the adverse events are likely to be related to the GMSCs in the trial. At this time, there may be a major problem with the safety of stem cells, and the trial should be terminated in time;

2) If external information (such as other high-quality studies or evidence) proves that the intervention is ineffective or effective, there is no need to continue the current clinical trial;

10. Efficacy and Safety Evaluation

10.1 Primary endpoint

The primary endpoint is the change of β-cell function from baseline assessed at week 12 (the first visit after 4 infusions). Subjects are provided a mixed-meal, and C-peptide levels are observed within 2 hours after food intake (0, 30, 60 and 120 minutes). The fasting C-peptide, peak C-peptide and the area under the curve (AUC) of C-peptide calculated by formula are used to assess the β-cell function.

10.2 Secondary endpoints

The secondary endpoints include:

a) Change of β-cell function from baseline assessed at week 18, 24, 36, 48 (the scheduled visits during follow-up period). Subjects are provided a mixed-meal, and C-peptide levels are observed within 2 hours after food intake (0, 30, 60 and 120 minutes). The fasting C-peptide, peak C-peptide and the AUC of C-peptide calculated by formula are used to assess the β-cell function;

b) Exogenous insulin demanded quantity change assessed at week 3, 6, 9, 12, 18, 24, 36, 48 (the scheduled visits throughout the trial);

c) Glycometabolism index assessed at scheduled visits throughout the trial:

- Hemoglobin A1c (HbA1c) levels at week 12, 24, 36, 48 (the scheduled visits every 12 weeks throughout the trial);
- Fasting blood glucose (FBG) assessed at week 3, 6, 9, 12, 18, 24, 36, 48 (the scheduled visits throughout the trial);
- 2h-postprandial blood glucose (PBG) assessed at week 3, 6, 9, 12, 18, 24, 36, 48 (the scheduled visits throughout the trial);
- The AUC of blood glucose at week 12, 18, 24, 36, 48 (the scheduled visits during follow-up period). Subjects are provided a mixed-meal, and blood glucose levels

are observed within 2 hours after food intake (0, 30, 60 and 120 minutes). The AUC of blood glucose are calculated by formula.

- Time in range (TIR) assessed at week 3, 6, 9, 12, 18, 24, 36, 48 (the scheduled visits throughout the trial). TIR measures the percentage of time a person's glucose levels stay within target range (3.9-10.0 mmol/L).
- Standard deviation of blood glucose (SDBG) assessed at week 3, 6, 9, 12, 18, 24, 36, 48 (the scheduled visits throughout the trial). SDBG quantifies glucose variability by calculating the standard deviation of blood glucose over a day.
- Coefficient of variation (CV) assessed at week 3, 6, 9, 12, 18, 24, 36, 48 (the scheduled visits throughout the trial). CV measures relative glucose variability by calculating the percentage ratio of standard deviation (SD) to mean blood glucose.
- Largest amplitude of glucose excursion (LAGE) assessed at week 3, 6, 9, 12, 18, 24, 36, 48 (the scheduled visits throughout the trial). LAGE quantifies the single most extreme glucose swing within 24 hours by calculating the difference between the highest and lowest blood glucose.

10.3 Exploratory endpoints

The exploratory endpoints include immunological and inflammatory indicators, oxidative stress markers, diabetic complication evaluation. Exploratory analyses of these endpoints will be performed using collected biological samples (including plasma, urine and isolated peripheral blood mononuclear cells) and on a case-specific basis. These may include single-cell RNA sequencing, flow cytometry, omics profiling and other advanced analyses. The final exploratory analyses will be determined based on actual situation.

10.4 Safety variables

Patient safety will be assessed through the collection of reported adverse events (AEs) and clinical laboratory data.

10.4.1 Adverse events (AEs)

Any adverse event that occurs to a subject during the course of the trial (from the date of signing an informed consent form to the last follow-up) is defined as an adverse event (AE), regardless of whether it is related to the treatment.

Adverse events of special interest (AESI) are AEs (serious or non-serious) required to be monitored, documented, and managed in a pre-specified manner. The AESI of this trial is hypoglycemia event, which will be documented using dedicated tick boxes when a subject suffered from any of the following situation:

- Severe hypoglycemia: events requiring active administration of carbohydrates, glucagon, or other resuscitative actions by another person. Blood glucose confirmation is not mandatory.
- Symptomatic hypoglycemia: there are typical symptoms of hypoglycemia, and blood glucose $\leq$ 3.9 mmol/L.
- Suspected symptomatic hypoglycemia: there are symptoms of hypoglycemia, but no blood glucose measurement has been performed.

- Asymptomatic hypoglycemia: there are not typical symptoms of hypoglycemia, but blood glucose ≤ 3.9 mmol/L.
- Pseudohypoglycemia: there are symptoms of hypoglycemia, but blood glucose > 3.9mmol/L.

Severe hypoglycemia, symptomatic hypoglycemia, suspected symptomatic hypoglycemia and asymptomatic hypoglycemia will be enrolled in hypoglycemia adverse event analysis, while pseudohypoglycemia will be excluded.

10.4.2 Laboratory data related to safety evaluation

a) Hepatic and renal function assessed at week 3, 6, 9, 12, 18, 24, 36, 48 (the scheduled visits throughout the trial), including alanine aminotransferase (ALT), aspartate aminotransferase (AST), creatinine, blood urea nitrogen (BUN), uric acid (UA).
b) Blood lipids assessed at week 12, 24, 36, 48 (the scheduled visits every 12 weeks throughout the trial), including total cholesterol, triglyceride, low-density lipoprotein cholesterol and high-density lipoprotein cholesterol.

10.5 Observation Period

48 weeks.

10.6 Background Information on the Safety of the Stem Cell

In the experimental study of the preparation of T1DM mouse model and cell transplantation, the suspension of GMSCs was injected intraperitoneally. After 10 weeks, HE staining was performed on lung, liver, spleen, and kidney tissue sections to check for any abnormal lesions. The results showed no abnormalities in the organs. Although there are no significant safety issues based on the current data, there is a lack of sufficient human study data, which means potential safety concerns cannot be ruled out. Other clinical trials of stem cell therapy have reported that patients may experience fever and headache, with symptoms typically resolving within 24 to 48 hours.

In addition, we invited a third-party safety evaluation center to evaluate the safety of stem cell preparations. GMSCs are safe and reliable in safety evaluation tests in mice or monkeys. The results can be seen in our preclinical research treatment.

11. Requirements for Recording Adverse Events and Methods and Measures for Reporting and Handling Serious Adverse Events

11.1 Adverse Events

11.1.1 Adverse Event Determination

Any adverse medical event that occurs to a subject during the course of a clinical trial (from the date of signing an informed consent form to the last follow-up) is defined as an adverse event, regardless of whether it is related to the trial product.

11.1.2 Handling Adverse Events

If the subject has an adverse event during hospitalization, it will be handled according to the following procedures:

After the occurrence of adverse events in subjects, researchers should deal with them in time, determine the severity of the adverse event and its relevance to the experimental stem cell preparation, and provide further treatment advice:

① General adverse events: the outcome of the event can be closely observed or appropriate symptomatic treatment can be carried out;

② Significant adverse events: the investigator should timely inform the principal investigator, and suspend treatment, adjust dose and give targeted treatment according to the condition of the subject. If necessary, the principal investigator may decide whether to disclose the blind urgently;

③ Serious adverse events: handle and report according to the following "Serious adverse events";

④ Th research physician will implement treatment according to the condition. If the damage of the subject exceeds the treatment capacity of the research department, the relevant department should be consulted and assisted in handling the case.

11.1.3 Record Adverse Events

① Researchers should truthfully fill in the adverse event record form, recording the subject of the adverse event, follow-up time, coding terms, study group, time of occurrence, severity, duration, relationship with treatment, measures taken and outcome.

② The occurrence, development, and treatment of SAEs should be meticulously documented in the original medical records and recorded on the CRF. The SAE should be tracked until it is properly resolved, the condition stabilizes, or the cause is identified. A final report must be submitted to the principal investigator, the ethics committee of the clinical research institution, the sponsor, the National Medical Products Administration (NMPA), and the National Health and Family Planning Commission.

11.2 Serious Adverse Events

11.2.1 Severe Adverse Event Determination

A serious adverse event is one of the following medical events that occurs during the clinical trial using any dose of the test drug: leading to death or life-threatening (here "life-threatening" means that the patient is at risk of death when a serious adverse event occurs, rather than assuming that a serious adverse event may cause death if it is more serious); requiring hospitalization or prolonged hospitalization; causing permanent or severe disability/loss of function; congenital malformation or birth defect; an event of important medical significance.

11.2.2 Handling of Serious Adverse Events

① When considering SAE, the attending physician shall notify the principal investigator or other responsible doctors to be present. If the condition is serious, the project leader shall be notified while emergency treatment is being given, and the trial injection shall be stopped immediately if necessary;

② If it is judged to be SAE, corresponding treatment or rescue measures should be taken immediately according to the clinical manifestations and the norms of clinical rescue treatment of each specialty to maintain the stability of the patient's vital signs as far as possible, and electrocardiogram monitoring should be carried out if necessary.

11.2.3 Reporting of Serious Adverse Events

In the event of a serious adverse event during clinical trials, researchers must immediately take necessary measures to ensure the safety and rights of the participants. Regardless of whether the adverse event is related to the trial product, researchers should report it to the relevant provincial, autonomous region, or municipal (food) drug administration, the National Medical Products Administration, and the sponsor within 24 hours. They should also promptly inform the ethics committee and other researchers involved in the same clinical study. If the serious adverse event is determined to be related to the trial product, the sponsor is responsible for the costs of emergency treatment and patient care, as well as providing appropriate economic compensation.

Researchers must complete the Serious Adverse Event Report Form and record in the original data when, how, and to whom serious adverse events will be reported. The sponsor ensures that all reporting procedures required by laws and regulations are met.

11.2.4 Follow-up of Serious Adverse Events

In the event of a serious adverse event, the research center must follow up the subject until full clinical recovery and laboratory test results return to normal, or until the condition stabilizes. This may mean that the follow-up continues even after the subject has completed the clinical study, and the monitoring team may request additional studies. The follow-up information must be recorded in the Serious Adverse Event Follow-up Form and submitted to the relevant monitor.

If a serious adverse event that has attracted the attention of the investigator occurs at any time after the study is started and the investigator believes that it is likely to be caused by the study, it should be reported immediately to the monitoring team.

11.3 Degree of Adverse Event Judgment

When filling out the adverse event form, the severity of the event is graded as follows:

Mild: symptoms appear but not affect normal life. And no symptomatic treatment or drug withdrawal is required.

Moderate: symptoms appear and have mild effect on normal life. And no symptomatic treatment or drug withdrawal is required.

Severe: symptoms appear and affect normal life, or symptomatic treatment or drug withdrawal is required.

Life-threatening: symptoms are severe, life-threatening, fatal or disabling, and the drug needs to be stopped immediately or emergency treatment is required.

Death: subject die.

11.4 Evaluation of the Correlation Between Adverse Events and Trial Product

According to the 'Manual for Reporting and Monitoring of Adverse Drug Reactions' issued by the NMPA and the 'Measures for the Reporting and Monitoring of Adverse Drug Reactions' released on July 1, 2011, researchers should conduct a comprehensive analysis of the specific circumstances of adverse events experienced by subjects, including their medical history, comorbidities, and concurrent medications, to determine the relationship between the adverse events and the drugs.

The five principles for evaluating the association between drugs and adverse events are: ① Is there a reasonable temporal relationship between the use of the drug and the occurrence of the adverse event? ② Does the event match the known adverse event types of the drug? ③ After discontinuing or reducing the dosage of the drug, does the event disappear or alleviate? ④ When the suspected drug is used again, does the same event reappear? ⑤ Can the event be explained by the effects of concurrent medications, the progression of the patient's condition, or the impact of other treatments? Correlation evaluation: Based on the above five principles, the association is divided into six levels: definitely relevant, probably relevant, might be relevant, probably irrelevant, to be evaluated and unable to be evaluated.

Judgment indicators / Judgment results	1	2	3	4	5
It is definitely relevant	+	+	+	+	-
It is probably relevant	+	+	+	?	-
It might be relevant	+	±	±?	?	±?
It probably irrelevant	-	-	±?	?	±?
To be evaluated	Additional information is needed for evaluation				
Unable to be evaluated	The necessary information for evaluation could not be obtained				

Note: + means yes; - means no; ± means difficult to be sure or deny; ? means unknown

11.5 Follow-up of Adverse Event Outcomes

Adverse events that have reached the study endpoint or have not yet been resolved must be followed up until one of the following events occurs:

① The event resolves;

② The event stabilizes;

③ If the baseline value is acceptable, the event returns to the baseline level;

④ When no other information is available (when the subject or the person caring for his/her health refuses to provide additional information, and the subject proves that follow-up efforts have failed).

12. Case Report Form

12.1 Data Record

1. CRF record requirements

The researchers fills in the CRF while diagnosing and treating the subjects to ensure that the data is recorded timely, completely, accurately and truthfully.

When any correction is made to the CRF, only a cross out line should be drawn and the revised data should be annotated by the investigator. The investigator should sign (sign the phonetic abbreviation of the investigator's name) and indicate the date, and the original record should not be erased or covered.

During the subject screening stage, physical examination is conducted. If the subjects have undergone several items similar to the screening test items recently (within 2 days prior to enrollment), they can be retained as screening test items after evaluation and confirmation by the investigator, and the relevant test results can be filled in the corresponding record form.

The original laboratory test sheets of inpatients should be attached to the medical records, and their laboratory results should be filled in the corresponding record sheet of the CRF.

2. Requirements for writing inpatient medical records

Researchers should fill in the CRF at the same time as filling in the inpatient medical record, and ensure that the contents of the CRF are consistent with those in the inpatient medical record.

3. Review of CRF

After the observation of each subject is completed, the investigator should timely fill in the relevant data on the CRF and submit it to the principal investigator for review and signature confirmation.

12.2 Data Monitoring

1. The monitoring of the trial is conducted in accordance with the protocol.

The sponsor appoints qualified monitors to conduct regular on-site comprehensive inspections to ensure strict adherence to the research protocol. Monitors regularly verify the informed consent forms and screening inclusion status of participants; confirm that all CRFs (CRFs) are correctly filled out and consistent with the original data; ensure that all errors or omissions have been corrected or noted, signed by the investigator, and dated; confirm and record any changes in dosage, treatment, concomitant medications, incidental diseases, missed visits, or missed examinations for each participant; verify that the withdrawal and missed visits of enrolled participants are explained in the CRFs; confirm that all adverse events have been recorded, and serious adverse events are reported and documented within the specified time frame; verify that the trial products are supplied, stored, distributed, and recovered in accordance with relevant regulations, with corresponding records kept.

When tracing the original medical records, the following points should be paid attention to:

① The name, dose and method of use of the test product shall be recorded in the original medical record.

② Do not use similar products that are prohibited by the clinical study protocol.

③ The items observed and examined and the records are consistent with the CRF.

④ Check whether the severity of the disease, the judgment of efficacy and the safety evaluation are correct.

⑤ The name, mailing address and hospital number of the subject in the medical record must be filled in and guaranteed to be true.

2. Transfer of research materials

The monitor shall sign and confirm the completed CRF after checking and verifying it, and submit it to the clinical research data administrator in time. For the transmission of completed CRFs among investigators, monitors and data administrators, there shall be special records. When received, there should be a corresponding signature, and the record should be properly preserved.

12.3 Data management

1. Data traceability, filling and handing over of CRFs (CRF)

The most original record is the original medical record, which needs to be properly preserved. The data of the CRF are all derived from the original medical record and filled in by the researcher. Each case selected to obtain a random number must complete the CRF. The completed CRF is reviewed by the clinical monitor and handed over to the data manager for data entry and management.

2. Database design and establishment

Establish a clinical research database to record all the information in the CRF form. The format of the database will correspond to the format of the CRF table as much as possible to facilitate data entry.

3. Data collection and modification

To ensure the accuracy of numerical data, Epidata3.1 is used for double-check data collection by two individuals. After the data collection is completed, a consistency check and statistical mechanism are conducted. For questions in the CRF, the data administrator will fill out the Data Review Question (DRQ) form and send inquiries to the researcher through the clinical monitor. The investigator should promptly respond and return the answers. The data administrator will then modify the data based on the investigator's responses, confirm the changes, and re-enter the data. If necessary, the DRQ can be sent again.

4. Data verification

Data verification will be conducted through manual and system checks. Manual verification involves data administrators identifying errors by checking the consistency and logic of the data, resulting in a DRQ. System verification, or computerized edit check refers to the verification of data in CRF by computer program methods, including scope, logical relationship, consistency, violation and deviation of the protocol, etc. The DRQ generated will be submitted to the clinical monitor for further review. The monitor hands it over to the investigator for reconfirmation. The relevant modifications need to be signed and dated by the investigator.

5. Data locked

When all of the following conditions are met, the data can be locked.

① All data have been collected into the database and double-checked.

② All questions have been resolved.

③ The analysis population has been defined and judged.
