# Supplementary material for: Safety and efficacy of human gingiva-derived mesenchymal stem cells in patients with type 1 diabetes: a double-blind, randomized, placebo-controlled, phase 1 trial
Source: Signal Transduct Target Ther. 2026 Sep 30;11:434. doi: 10.1038/s41392-026-03021-8 (PMC13627707; doi:10.1038/s41392-026-03021-8)
Supplement: Supplementary file 3 [file 41392_2026_3021_MOESM1_ESM.docx]

Supplementary Materials for

Safety and Efficacy of Human Gingiva-Derived Mesenchymal Stem Cells in Patients with Type 1 diabetes: a double-blind, randomised, placebo-controlled, phase 1 trial.

Qi Tang, Li Zhou, Liwen Fan, Wei Zhang, Xixiang Tang, Donglan Zeng, Mei Li, Yanhua Zhu, Mengyin Cai, Jia Yuan, Zheng Chen, Song Guo Zheng, Yanming Chen

Correspondence to: [Song.Zheng@shsmu.edu.cn](mailto:Song.Zheng@shsmu.edu.cn) and chyanm@mail.sysu.edu.cn

**This PDF file includes:**

Materials and Methods

Supplementary Figure 1 to 7

Supplementary Table 1 to 3

Materials and Methods

1. **Inclusion and exclusion criteria for patients**

**Inclusion criteria**

a. Aged from 18 to 60 years old without sex limitation.
b. Signed informed consent form;
c. Mentally normal and able to comply with the procedures of the trial protocol;
d. Meets the WHO 1999 diagnostic criteria for type 1 diabetes mellitus (T1DM);
e. Duration of T1DM ≤ 10 years at enrollment;
f. Baseline C-peptide level ≤ 0.3 ng/mL;
g. Women with childbearing potential must have no pregnancy plans within 1 year.

**Exclusion Criteria**

Subjects meeting any of the following criteria will be excluded from the study:

a. Body mass index (BMI) >30 kg/m²;
b. Daily insulin requirement >100 U/day;
c. Aspartate aminotransferase (AST) or alanine aminotransferase (ALT) >2.5 times the upper limit of normal and/or serum creatinine (Cr) ≥133 μmol/L (1.5 mg/dL);
d. Severe hepatic or renal dysfunction, proliferative diabetic retinopathy, or macular edema;
e. History of coronary angioplasty, coronary stent implantation, coronary artery bypass grafting, myocardial infarction, unstable angina, clinically significant ECG abnormalities, cerebrovascular accident, or transient ischemic attack;
f. Acute or chronic pancreatitis; active infections (including hepatitis C, HIV, or tuberculosis confirmed by positive skin test or clinical manifestations), or suspected tuberculosis requiring treatments;
g. Long-term systemic corticosteroid therapy; bleeding disorders (e.g. hemophilia, idiopathic thrombocytopenic purpura);
h. Malignancy, psychiatric disorders, or hypersensitivity to mesenchymal stem cells (MSC) or preservatives;
i. Pregnant or breastfeeding women;
j. Other severe comorbidities deemed unsuitable for inclusion by the investigator.
2. **GMSCs characterization identification**
   2.1 Morphological criteria:

Under microscopic observation at 40× and 100× magnification, cells exhibit a relatively uniform spindle shape, grow in a vortex pattern, and have good transparency.

2.2 Cell number and cell viability:

Trypan blue staining was used to count the number of dead and live cells, and cell viability was calculated therefrom. The viability of qualified cells should be ≥ 90%.

2.3 Characterization analysis:

The phenotypic characterization of GMSCs were confirmed using flow cytometry. A panel of antibodies (BioLegend) targeting CD90 (#328114), CD105 (#323208), CD73 (#344016), CD45 (#304038), CD34 (#343606), CD19 (#392504), HLA-DR (#307606), CD11b (#393103), CD86 (#305406), CD80 (#375405), CD44 (#397503), CD29 (#303003), and CD45 (#304012) were used to detect the cell surface markers. The proportions of CD90^+^, CD105^+^, and CD73^+^ cells should be ≥ 95%, and the proportions of CD45^+^, CD34^+^, CD11b^+^, CD19^+^, and HLA-DR^+^ cells should not exceed 2%.

2.4 Evaluation of cell differentiation capacity:

GMSCs have differentiation potential to osteoblasts, adipocytes and chondroblasts under standard *in vitro* differentiating conditions.

3. **Randomization and Blinding**

The study adopted a block randomization design (block size = 4). Random number sequences were generated by statistical professionals using SPSS 24.0 software and were assigned to Group A and Group B in a 1:1 ratio. Allocation concealment was implemented via sequentially numbered, opaque, sealed envelopes, which were secured by researchers at the Zhongshan Ophthalmic Center of Sun Yat-sen University who were solely responsible for preparing the stem cell and placebo infusions and were not involved in patient recruitment, intervention, follow-up, or data analysis.

Upon enrollment at the Third Affiliated Hospital of Sun Yat-sen University, researchers at the Zhongshan Ophthalmic Center opened the envelope in the order of enrollment to confirm each patient's group assignment, with strict prohibition of number skipping. Both the researchers at the Third Affiliated Hospital and the enrolled patients remained blinded to the group assignments throughout the study.

During each patient's hospitalization, researchers from the Zhongshan Ophthalmic Center prepared either the stem cell infusion or placebo based on the patient's group assignment. Only the researchers preparing the infusions knew the specific treatment corresponding to group A and B. Once preparation was finished, the infusions was delivered to the Third Affiliated Hospital for clinical intervention. The gingival mesenchymal stem cells (GMSCs) mixture contained GMSCs at a dose of 1.0×10^6^ cells per kilogram of body weight, suspended in 200ml of normal saline. The placebo consisted of 200ml of normal saline. All preparations were packaged in brown light-resistant infusion bags, and the bags were immediately retrieved and returned to the Zhongshan Ophthalmic Center after each infusion. Neither the clinicians nor the patients could distinguish between the preparations based on factors such as dosage form, appearance, odor, volume, or packaging.

A two-stage unblinding process was conducted after database lock. The first unblinding allowed the statistical analysts to receive the patient group labels (Group A or Group B) to perform inter-group difference comparisons. Following the completion of statistical analyses, the second unblinding was carried out to reveal the specific treatment corresponding to Group A or Group B.

4. **Statistical Analysis Methods**

**Wilcoxon rank-sum test**

Data including the area of C-peptide levels under the concentration-time curve (AUC), peak C-peptide levels, fasting C-peptide levels, daily insulin dose, HbA1c, islet cell antibody, insulin autoimmune antibodies, glutamic acid decarboxylase antibody, insulinoma associated-2 antibody, alanine aminotransferase, urinary albumin to creatinine ratio, uric acid and flow cytometry data were non-normally distributed, and were analyzed using Wilcoxon rank-sum test, with the results expressed as median and interquartile range (IQR). Since the lower limit of the detection range for C-peptide levels at our research center was 0.02 nmol/l, when a patient's C-peptide level fell below this detection limit, the minimum value of 0.02 nmol/l was assigned and included in the statistical analysis.

**T test**

Data including age, weight, BMI, duration, fasting blood glucose, two-hour postprandial blood glucose, the area of blood glucose under the concentration-time curve (AUC), islet cell antibody, aspartate aminotransferase, creatinine, blood urea nitrogen, total cholesterol, triglyceride, low-density lipoprotein cholesterol, high-density lipoprotein cholesterol and 24-hour urine albumin were normally distributed with homogeneous variance, therefore T test was employed for between-group comparisons, with the results expressed as Mean±SD.

**Chi-square test**

For categorical data including sex and adverse events, Fisher’s exact test was used for analysis.

The *P* value was two-sided for all end points and a *P* value of less than 0.05 was considered to indicate statistical significance.

Type or paste text here. You can break this section up into subheads as needed (e.g., one on “Materials” and one on “Methods”).

Supplementary Figure 1. Study design and CONSORT flowchart.

(a) Study design. (b) Participant enrollment flowchart.

Supplementary Figure 2. Fold changes of C-peptide levels from baseline.

Fold changes from baseline of (a) area under the C-peptide curve, (b) fasting C-peptide, (c) peak C-peptide level. The data are represented as the mean ± SEM. *P<0.05. **P<0.01.

Supplementary Figure 3. Glucose metabolism.

Dynamic follow-up of the (a) fasting blood glucose, (b) postprandial blood glucose, (c) area under the blood glucose curve, (d) glycosylated hemoglobin, type A1C, (e) time in range, (f) SD of blood glucose, (g) coefficient of variation, (h) largest amplitude of glucose excursion in GMSCs group and placebo group during the study. (i) The fold change of autoantibodies at week 48 from baseline. The data are represented as the mean ± SEM. IAA, insulin autoantibody; ICA, islet cell antibody; GADA, glutamic acid decarboxylase antibody; IA2, insulinoma-associated antigen 2 antibody.

Supplementary Figure 4. Single-cell RNAseq of total PBMCs and subpopulation analysis of CD4^+^ T cells.

(a) UMAP visualization of single cells of total PBMCs in gene expression space. Colors represented different groups. (b) Stacked bar graph showed the percentage of each cluster in total PBMCs. (c) UMAP visualization of single cells of CD4^+^ T cell subpopulation. Clustering identified 5 major clusters: effector CD4^+^ T cluster 1; effector CD4^+^ T cluster 2; central memory CD4^+^ T; effector memory CD4^+^ T; Treg, regulatory CD4^+^ T. (d) Marker genes of the 5 clusters in (c). (e) The proportion of each cluster in CD4^+^ T cell subpopulation. The data are represented as the mean ± SEM. (f) Heatmap showed the expression of key genes in Treg cell subpopulation based on single-cell RNAseq data. (g) Intercellular communications among celltypes assessed by Cellchat (ver 2.1.2). The arrow lines connected the signal-sending cells and signal-receiving cells. The thickness of the lines represented the strength of intercellular communications. ns, not significance.

Supplementary Figure 5. Subpopulation analysis of monocytes and NK cells.

(a) UMAP visualization of single cells of monocytes cells in gene expression space. 3 clusters were identified: cMo, classical monocytes; iMo, intermediate monocytes; ncMo, non-classical monocytes. (b) Marker genes of the 3 clusters in (a). (c) Proportion of the monocytes subpopulations. The data are represented as the mean ± SEM. (d) The expression levels of inflammatory cytokines and functionally critical genes in monocytes subsets. (e) UMAP visualization of single cells of NK cells in gene expression space. Clustering identified 2 major clusters: CD56^dim^ and CD56^bright^ NK cells. (f) Marker genes of the 2 clusters in (e). (g) Proportion of the NK cells subpopulations. Data are represented as mean ± SEM. (h) The expression levels of inflammatory cytokines and functionally critical genes in NK cells subsets. ns, not significance.

Supplementary Figure 6. Subpopulation analysis of CD8^+^ T cells.

(a) UMAP visualization of single cells of CD8^+^ T cells in gene expression space. Colors represented the exhaustion score calculated by Addmodulescore. (b) Exhaustion scores of the 5 identified clusters. (c) Pseudotime analysis of CD8^+^ T cell. (d) Exhaustion scores of total CD8^+^ T cells in different groups. (e) Intercellular communication based on receptor-ligand interactions by Cellchat (ver 2.1.2) in GMSCs group. The receptor-ligand pairs were displayed in rows and signal sending-receiving cell pairs in columns. Each plot represented the communication probability of the corresponding receptor-ligand signal in the cell pairs.

Supplementary Figure 7. The expression levels of exhaustion-associated markers in CD8^+^ T cells by single-cell sequencing analysis.

**Supplementary** Table 1. The glycemic level of the patient who achieved insulin withdraw.

|  | **V1** | **V2** | **V3** | **V4** | **V5** | **V6** | **V7** | **V8** | **V9** |
| --- | --- | --- | --- | --- | --- | --- | --- | --- | --- |
| Daily insulin dose, IU/d/kg | 0.31 | 0 | 0 | 0 | 0 | 0 | 0 | 0 | 0 |
| Fasting C-peptide, nmol/l | 0.14 | - | - | - | 0.16 | 0.22 | 0.22 | 0.2 | 0.12 |
| Peak C-peptide, nmol/l | 0.29 | - | - | - | 0.62 | 0.5 | 0.43 | 0.51 | 0.51 |
| AUC of C-peptide, nmol/l*h | 0.47 | - | - | - | 0.82 | 0.82 | 0.73 | 0.72 | 0.56 |
| HbA1c, % | 13.2 | - | - | - | 6.1 | - | 6.8 | 6.6 | 7.8 |
| GA,% | 45.3 | 18.6 | 18.6 | 19 | 18.2 | 19.9 | 22.7 | 20.8 | 26.8 |
| FBG, mmol/L | 6.77 | 5.37 | 5.74 | 5.91 | 6.06 | 6.66 | 7.00 | 7.04 | 7.71 |
| 2h-PBG, mmol/L | 8.71 | 6.82 | 7.56 | 7.96 | 7.71 | 8.06 | 10.60 | 10.01 | 12.92 |
| IAA, COI | 0.07 | - | - | - | - | - | - | - | 0.09 |
| ICA, COI | 64.5 | - | - | - | - | - | - | - | 61 |
| GADA, IU/ml | >2000 | - | - | - | - | - | - | - | >2000 |
| IA2, IU/ml | 1.86 | - | - | - | - | - | - | - | 1.51 |

IAA, insulin autoantibody; ICA, islet cell antibody; GADA, glutamic acid decarboxylase antibody; IA2, insulinoma-associated antigen 2 antibody.

**Supplementary Table 2. Clinical parameters after GMSCs/Placebo treatment.**

| **Index** | **Visit 5** | | | **Visit 9** | | |
| --- | --- | --- | --- | --- | --- | --- |
|  | **Placebo** | **GMSC** | ***p*** | **Placebo** | **GMSC** | ***p*** |
| Weight, kg | 62.43±10.14 | 57.76±6.35 | 0.319 | 60.58±6.68 | 60.34±3.50 | 0.946 |
| BMI, kg/m^2^ | 23.52±1.91 | 20.73±2.70 | 0.037 | 23.80±1.10 | 21.64±2.06 | 0.084 |
| AST, U/L | 22.75±9.74 | 19.11±5.67 | 0.374 | 23.57±13.70 | 15.33±3.16 | 0.166 |
| ALT, U/L | 13 [11, 19.5] | 13 [12, 15] | 0.770 | 17.43±9.69 | 13.00±2.78 | 0.281 |
| Cr, umol/L | 56.50±7.96 | 53.67±13.17 | 0.596 | 52.62±10.62 | 58.43±13.18 | 0.380 |
| BUN, mmol/L | 4.62 [4.13,6.07] | 4.1 [3.90, 5.41] | 0.815 | 4.56 [4.37, 5.09] | 5.79 [4.84, 5.99] | 0.573 |
| TG, mmol/L | 0.61 [0.59,0.80] | 0.61 [0.5, 0.67] | 0.386 | 0.83 [0.77, 1.11] | 0.54 [0.50, 0.88] | 0.368 |
| TC, mmol/L | 4.98±1.14 | 4.60±0.64 | 0.426 | 4.15 [3.85, 4.70] | 3.99 [3.93, 4.65] | 1.000 |
| HDL, mmol/L | 1.73±0.40 | 1.55±0.21 | 0.270 | 1.42±0.30 | 1.32±0.36 | 0.555 |
| LDL, mmol/L | 2.62±0.99 | 2.41±0.60 | 0.615 | 2.20 [1.88, 3.02] | 2.54 [2.18, 2.62] | 0.918 |
| UA, mmol/L | 340.0±69.8 | 321.0±111.2 | 0.676 | 285.5±24.3 | 366.3±93.3 | 0.046 |

**Supplementary Table 3. Principles of daily insulin dose adjustment.**

| **Basal insulin adjustment^a^** | |
| --- | --- |
| **Fasting glucose level** | **Adjustment** |
| <3.1 mmol/L | -4U |
| 3.1-4.0 mmol/L | -2U |
| 4.0-6.1 mmol/L | No adjustment |
| 6.2-7.8 mmol/L | +2U |
| 7.9-9.9 mmol/L | +4U |
| ≥10 mmol/L | +6U |

^a^ Bonus insulin was adjusted for individual insulin to carbohydrate ratio (ICR) and/or insulin sensitivity factor (ISF) and target glucose.
